# Ninth International Medical Congress

**Published:** 1887-11

**Authors:** 


### NINTH INTERNATIONAL MEDICAL CONGRESS.

HELD IN WASHINGTON, D. C., SEPTEMBER 5, 6, 7, 8, 9, AND 10, 1887.

[CONTINUED FROM THE OCTOBER NUMBER.]

### GENERAL SESSION.

### FIFTH DAY.

The Congress was called to order punctually at 10 A. M., by the President.

Secretary-General, Dr. Hamilton, reported that the committee appointed on the time and place for holding

THE TENTH INTERNATIONAL MEDICAL CONGRESS, had recommended Berlin, Germany, as the place and the year 1890 as the time.

The report was unanimously adopted.

Dr. Hamilton also reported resolutions adopted by the Section in Military and Naval Surgery and Medicine, the purport of which was a recommendation of

UNIFORMITY OF THE REPORTS OF SICK AND WOUNDED IN ALL THE ARMIES OF THE WORLD.

Dr. Hamilton further reported as having been adopted by the Section in Public and International Hygiene, the following preamble and resolution :

*Whereas*, The whole community has been shocked by the almost daily occurrence of terrible accidents on many of the railroads, causing considerable loss of life, and by the habitual neglect of the most elementary sanitary laws ;

*Whereas*, As this Section considers itself in a degree the guardian of public health ; be it

*Resolved*, That the attention of this Ninth International Medical Congress be respectfully called to this most important question, and that it be requested to use its influence to obtain the necessary reforms.

The following were reported from the same section on *Thursday :*

After the reading by Dr. Domingo Freire, of Rio Janeiro, representative in the Congress of the Brazilian Government, of a paper entitled "Vaccination with the Attenuated Culture of the Microbe of Yellow Fever," with demonstration of the Microbe under the microscope, the following preamble and resolutions were adopted by the section :

*Whereas*, Inoculation against yellow fever, if it proves successful after further examination, is calculated to benefit the human race throughout the world ; and

*Whereas*, The facts presented by experiments of Dr. Domingo Freire afford a reasonable assurance of its protective influence in Rio Janeiro; therefore,

*Resolved*, That this section recommends the co-operative investigation of the results obtained by yellow fever inoculations as a protective against that disease, and that adequate appropriations by the governments represented in this Congress be made for that purpose.

*Resolved*, That this action be communicated forthwith for consideration in the general session of the Congress.

The president then called upon Dr. Charles D· C. Phillips, of London, England, to occupy the chair during the delivery of the general address by G. Fielding Blandford, M. D., London, England, on

THE TREATMENT OF RECENT CASES OF INSANITY IN ASYLUMS AND IN PRIVATE HOUSES.

The inquiry was limited to the treatment of recent insanity only, without reference to the care and maintenance of the chronic insane. Recent insanity in very many cases is a curable disorder. What mode of treatment, then, will afford the best prospect of recovery? Will the latter take place only under asylum supervision, or is there a fair chance of curing the sufferer in a house taken for the purpose, or the house of a medical man? For the great majority of patients asylum treatment is the only available method, but there is a certain proportion of insane persons whom it is important to treat, if it be possible, outside an asylum, to save them from the stigma of having been the inmates of one, as this may seriously damage their future prospects and position. Among females some may be of high rank, whose friends shrink from the publicity of certificates and asylum surroundings; some may be young girls at the outset of life; others young mothers whom and whose children it is desirable to save from the reminiscence of an asylum. Among men there are not a few to whom confinement in an asylum will certainly bring a loss of employment tantamount to ruin. And a young man's future career may be marred by the same fact. It is, therefore, of importance to consider what mental disorders are likely to be transitory and capable of being treated in an ordinary house, or slight and free from danger to the patient and those about him, so that he may be able to live in such a house, or move from place to place under the charge of suitable companions.

The most transitory of all mental disorders are attacks of maniacal excitement, varying under undue elation and hilarity to very acute mania. The most frequent cases are those caused by drink varying from ordinary delirium tremens to other forms of insanity seen more especially in women. Very similar forms occur without any history of drink, in persons prone by inheritance to nerve disturbance. They arise after a sudden shock of bad, or even good, news, or great fatigue, or other exhausting cause, and last a few hours, days or weeks, the subsidence being often as rapid as the onset. The prognosis as regards recovery is often easy, the doubt being as to duration, and this will be solved by proper treatment.

Patients who are not actively excited or unduly elated, but depressed and melancholic, must be considered from a different point of view. They are more manageable, at any rate, in the less acute forms, but the disorder is more insidious, and treatment will have to continue for months rather than weeks or days. But many recover without asylum treatment, many without the aid of a specialist. As the commencement of this depression is almost always slow and insidious, treatment outside an asylum is likely to be adopted at first, so that there is an opportunity of considering how far such treatment is answering or or likely to answer. The first objection to be urged against the keeping of a melancholic patient in a private house is the danger of suicide. That an ordinary house with unguarded windows, staircases and fireplaces offers more facilities for suicide than an asylum is certain. But asylums do not afford absolute protection, as our commissioners' reports abundantly testify. Nothing will effectually prevent suicide but the unceasing vigilance of good attendants, and this, in many cases, can be applied as well out of as in an asylum. One symptom which often necessitates removal to an asylum is obstinate refusal of food. When this is determined, and forcible feeding requires the aid of several assistants, it can hardly be carried out in a private house. Another reason why an asylum may be preferred is, that in an ordinary house or family the intense egotism so characteristic of melancholica is fostered rather than removed by the concentration of the attention of all upon the patient and his delusions. In an asylum, especially a large one, he becomes a unit in the number of those treated, and this of itself is often highly beneficial. There is one more variety of insanity which can often be treated out of an asylum. This is acute primary *dementia*, or *stupor*, as it is sometimes called. These patients, though often very lost and idiotic, are seldom dangerous. With plenty of food, air, and exercise, they will get well in a private house and as they are almost all young persons, it is an advantage that they should escape the asylum reputation.

For the treatment of all patients in private houses one thing is absolutely requisite, viz., funds. Unless proper medical supervision, proper attendants, and proper quarters can be provided and paid for, to an asylum the sufferer should go. Friends are often willing to make a sacrifice, and profess they will pay anything. But this may mean a scanty and poverty stricken supply of what is necessary, the funds become exhausted before recovery takes place, and then an asylum is necessary, and money is wasted which could hardly be spared. Where means are slender and the duration of the illness doubtful, it is better to send the patient to an asylum at once, and save the money for the convalescent stage. Treatment in a patient's own house is rarely successful, therefore the alternatives are an asylum, or a house adapted to the requirements of the case.

At the close of the address, Dr. A. Cordes, of Geneva, Switzerland, moved a vote of thanks to Dr. Blandford for his able and interesting address, which was seconded by Dr. P. H. Kretzschmar, of Brooklyn, New York, and then unanimously adopted.

The congress then adjourned to meet at 9:30 A. M., on Saturday.

---

### SECTION ON GENERAL MEDICINE.

### Fifth Day—Morning Session.

Professor George E. Stubbs, of Philadelphia, Pennsylvania, read a paper entitled

RATIONAL TREATMENT OF DISEASES OF THE RESPIRATORY APPARATUS.

He made an elaborate and somewhat extended review of the subject of phthisis. Dust inhaled into the lungs was a great exciting cause of phthisis. In treating this disease he was partial to counter-irritation with moisture and heat. For this purpose he used flannel wrung out in hot mustard-water, applying this around the chest and putting a dry layer of flannel over it.

Dr. Thomas Hay, of Philadelphia, Pennsylvania, thought counter-irritation invaluable. He paints the whole exterior of the chest with iodine. He gives large doses of whiskey.

Dr. Ege, of Reading, Pennsylvania, read a paper on

A NEW METHOD OF TREATING PHTHISIS.

He does not believe in the cure of phthisis unless it can be demonstrated by the microscope. Whether the bacillus tuberculosis is, in itself, the cause of the phthisis, makes no difference so long as we know it to be the invariable accompaniment of this disease.

To the white of one egg are added six to eight ounces of water; this mixture is put away in a bottle for five or six days, until the mixture has the odor of rotten egg. From one-half to two ounces of this fluid are placed in an atomizer and deeply inhaled during twenty-four hours. Under this treatment the expectoration becomes thinner and of less quantity, the microscopic examination of the sputa shows a less number of the bacilli, and finally none at all. The experiments attempting to prove that the inhalations of the bacterium termo would exterminate the bacillus tuberculosis have proved a failure. While it is impossible to speak definitely upon the *modus operandi* of this solution, Dr. Ege is confident in regard to the favorable clinical results. He suggests also that, among the fifteen or twenty varieties of bacteria contained in the egg solution, there are probably some which have a fatal antagonistic action upon the bacillus of phthisis. He stops the treatment when the sputa no longer contain the bacilli.

Dr. Ege was asked what he thought of the sulphuretted hydrogen treatment, to which he replied that it relieved, but did not cure, phthisis.

Sir James Grant, of Canada, read a paper on

DIPHTHERIA.

Diphtheria is of two varieties—the simple and the malignant. We must look upon this disease as a blood poison. He has used mustard baths at the beginning of the attack, containing one ounce of mustard to an ordinary bath-tub of water, after which the patient is wrapped in blankets and diaphoresis encouraged. He uses light treatment, iron internally and applied to the pharynx. Nourishment should be encouraged.

We must be very careful about letting the patient move about suddenly, on account of the danger of paralysis of the heart. He must be kept long in the reclining position.

As to the malignant type of the disease, he had never seen but one epidemic, and that about twenty years ago. He knew of no remedies for this form of disease.

It was interesting to note that when there is a large snowfall and consequent dryness of the atmosphere, diphtheria is less prevalent, and *vice versa*. Moisture in the atmosphere has a predisposing influence. Cases usually do better inland than at the seashore.

Professor John A. Octerlony, of Louisville, Kentucky, remarked that he was exceedingly grateful to Sir James, especially for the valuable suggestions of diverting to the surface and eliminating through the perspiration the poison; it was an idea which he had never heard or thought of before.

Professor A. B. Palmer, of Ann Arbor, Michigan, said that he brought the system early under a free perspiration and salivation by means of jaborandi, taking into account, of course, the condition of the heart. He had used counter-irritation, with a similar idea to that of Sir James, in spinal meningitis.

Sir James Grant replied that he had never used jaborandi. Mustard had the advantage of being well known and handy.

---

### SECTION ON GENERAL SURGERY.

### FIFTH DAY—MORNING SESSION.

Dr. George Assaky, of Bucharest, Roumania, read a paper on

IODOL IN SURGERY.

The conclusions arrived at were: (1) Wounds unite under iodol by first intention. This union, however, being the result of various and complex conditions attending operation. It is not possible, therefore, to attribute to iodol alone the absence of suppuration and inflammatory conditions. In wounds which gap and suppurate iodol is an excellent antiseptic. It rapidly retards suppuration, renders it inodorous, reduces the necessity for frequent dressing, and hastens considerably cicatrization. In ulcerating or gangrenous wounds iodol aids in resisting the destructive process, and changes the wound, after a variable period of time, to a healthy, granulating condition. This action of iodol is shown, also, in hard chancres. In case of soft chancres the result is variable. Sometimes it transforms them into a simple wound with brief delay; at others it is insufficient for this purpose, and it becomes necessary to employ in addition, locally, antiseptic lotions.

The same is true with reference to open venerial buboes of the groin. The powdered iodol has this advantage over iodoform, that it is free from odor and is not toxic in its effects.

(2) Doses of iodol of from 0.40 centigrammes to 2.0 grammes, daily, produce no functional trouble, even if continued a long time.

These doses give marvelous results in tertiary syphilis and in scrofulous affections. In the secondary stage of syphilis, taken internally, it rapidly destroys the syphilitic manifestations. Iodol seems to aid the general nutrition and increase strength and flesh. It is indicated in all cases of specific malnutrition.

Iodol is an antipyretic. In acute infectious diseases, such as erysipelas, etc., it causes a rapid fall of temperature.

Dr. Milton J. Roberts, of New York, N. Y., read a paper on

### A NEW METHOD OF OPERATING ON BONE BY MEANS OF THE ELECTRIC OSTEOTOME.

He presented an apparatus for this purpose, of ingenious mechanism.

The osteotome proper consisted of a hollow cylindrical handle, protruding from which was the shaft of the instrument, at the end of which was a small wheel with teeth like an ordinary finger-saw; the base of the handle was connected with the electric batteries by means of the covered wires usually used in electric batteries; anterior to the handle were "cut offs," which could be worked by the forefinger and thumb disconnecting the current in an instant and checking the revolutions of the saw-wheel just as quickly; according to the diameter of the bone, different-sized saws were adjusted. In connection with this was a set of drills for drilling into any part of a diseased bone and removing the sequestrum or dead tissues in the bony cavity, the cavity being illuminated by a small electric light thrust in at different stages of the operation in order to secure removal of all carious bone. The femur was exhibited, the head of which had been drilled in this manner, the drill-opening into the bone being three-eighths of an inch in diameter, while the entire inside portion had been removed by the drill.

The question was raised as to the cost of such an apparatus. It might cost more than the surgeon would care to invest unless making a specialty of such diseases. One gentleman affirmed that he could perform all the work which this instrument did with the regular dental drill, and that he had done it on several occasions.

Dr. Close, of Illinois, thought the chief advantage of the apparatus was that the operator does not necessarily require skilled assistance, as the current was controlled by the hand in which the instrument was held, while the left hand was at liberty. He had been in the habit of using at times the Band wheel-engine for such purposes, but found it too slow; in order to cut bone the saw must revolve quickly in such an apparatus.

Dr. Roberts, in conclusion, said that he had tried the dental engine many times and found the revolutions too slow, and it was for that reason he had set to work in order to secure the best possible method of removing bone in such cases; when the wheel revolves slowly the bone-tissue is torn; had it been satisfactory, he would not have spent the time and money in bringing forward a new instrument. In operating, the periosteum is not lifted or removed, but cut through with the bone. The speaker also exhibited some instruments for measurement, which would record the exact size of the bone to be removed in cases of deformities, etc.

Dr. George E. Post, of Beirut, Syria, read a paper on

### CALCULUS IN SYRIA.

The writer remarked that it would almost seem to be produced from climatic causes, as it was such a prevalent malady there. In Palestine, in almost every village, these cases are numerous both in young and old; in one day four patients came to him from a single village. When an operation is performed, the stone is generally found to be larger than is found in European countries, owing to the fact that the natives are extremely averse to surgical operations, and hence procrastinate. There are not many good surgeons there, and they frequently hesitate to perform an operation for stone; besides, too, the people are ignorant and poor. In that region there is a class of men called "stone-cutters," who make it their business to travel and remove stone from the bladder. They carry a bag containing the stones they have removed, and also those bequeathed to them by their ancestors, if in that calling. Their method of operation is as follows: The patient is laid on his back, without the use of anæsthetics, and held by assistants; the stone-cutter inserts two fingers into the rectum and feels for the stone in the bladder; when felt it is pushed toward the neck of the bladder, and then, with the aid of a razor scalpel, he cuts down upon the stone through the perineum, and, when reached, the stone is pushed out by the fingers in the rectum.

The writer quoted one of his cases, in which five stones were removed, two from the bladder and three from the perineal tissue. Another case was more curious than that; the patient had been operated on in infancy by the method described, and a fistulous opening still existed. In that country they are in the habit of eating grapes with skin, seed and pulp; the fistulous opening connecting the rectum with the urethra had secreted grape-seeds, these forming the nucleus for the stone formation. There were sixty-four stones removed from the fistulous tract and bladder, ranging from the size of a grape-seed to a nut; a portion of one stone was imbedded in the prostate, and with a short neck the larger portion was imbedded in the bladder.

Another case of the same character, but much larger in size, weighing six ounces, in which a portion of the stone was in bladder and prostate, was removed, and not the slightest disturbance of the system ensued, although the stone was of such large size. In another instance the speaker removed a stone weighing twelve ounces; it was four inches long and two inches wide.

The largest number of calculi he had ever removed from one patient was two hundred of various sizes, but eleven of them weighed six ounces each. The patient died from double pneumonia three days after. The speaker stated that he had operated in 250 cases of stone in the bladder. The mortality was 10 in 176 cases. One hundred and six of the cases (250) were in children ten years of age and under. He had performed 44 lithotrities, 8 of which died. One patient, a man seventy years of age, came under the writer's care from whom he removed three stones from the urethra—one from the membranous portion; one, three inches from the meatus; and one from just inside the meatus—the largest was the size of a bean. Going from extreme age comes that of a child, not quite two years of age, from whom the writer had removed a stone the size of a bean.

Dr. J. A. S. Grant (Bey), of Egypt, remarked that calculus is very common there also; a friend of his, Dr. Zacharol, had made careful and scientific examinations of the various calculi, and almost invariably found the nucleus to be the egg of Bilharzia hæmatobia, and which, he suggests, would account for the stone.

A patient came to the speaker's office one morning with his scrotum greatly inflamed, and about the size of a child's head. The man—a native—while sitting near the railroad track had been struck on the scrotum by a bottle thrown from the hands of a soldier in a train that was passing; the blow had laid open the scrotum. The speaker removed the stone, which weighed fourteen ounces. This was the largest he had ever removed, and this was taken away in three pieces.

Dr. R. T. Morris, of New York, asked which species of Bilharzia was found, and also in how many cases the blood-worms were found.

Dr. Grant replied that Bilharzia hæmatobia was found in almost every case examined by Dr. Zacharol.

Dr. Post remarked that he hardly thought Bilharzia could be a cause, as while Bilharzia was very rare in Syria, calculi were very common.

Dr. Oscar J. Coscary, of Baltimore, Maryland, read a paper on

AN UNCOMMON CASE OF FRACTURE, WITH DISLOCATION OF THE METATARSUS.

The patient, a male, was run over by a steam-car, the right leg being crushed, compelling amputation, and the left foot cut. The patient died nine hours after the accident. At the autopsy it was found that three of the metatarsal bones were completely luxated, the scaphoid projecting up, presenting the appearance very much of such bone in a case of strong pes equinus, while the posterior portion of the os calcis had been crushed and the astragalus bruised.

Professor N. Senn, of Milwaukee, Wisconsin, read a paper on

ELASTIC CONSTRICTION OF THE NECK, WITH EXCLUSION OF THE TRACHEA, AS A MEANS OF CONTROLLING HŒMORRHAGE IN OPERATIONS ON THE HEAD.

He called attention to the fact that where we desired a bloodless operation there were more blood-vessels than in any other part of the body. He thought, however, this hæmorrhage could be overcome by the following method, which he had practiced on dogs, namely, cutting down on the trachea, separating it from the surrounding tissues, and then passing a strong rubber band behind it, the ends passing backward; the vessels of the neck could be constricted by tightly securing the ends of the rubber band, while respiration could be carried on. One dog, upon which he had operated, retained the bandage for two hours, and is now as well as ever. Another one, where it was retained still longer, had been put under the table, and afterward, when about to be thrown out, it was noticed the heart pulsated, but respiration had ceased. The band was released and artificial respiration practiced, when the animal speedily recovered.

Dr. Carnochan, of New York, N. Y., presented a specimen of

DOUBLE DISLOCATION OF THE HIP-JOINT.

He remarked that previous to 1820 this condition was unknown, but in 1826 Dupuytren first brought it to the notice of the profession. The speaker stated there were various theories as to the causation of this condition, among which were the carelessness of the accoucheur, movement of the fœtus in utero, etc.; the question arises, also, as to whether it is really a dislocation or malformation.

Dr. R. T. Morris, of New York, N. Y., stated that he did not think that it could be caused by careless handling of the physician, as in the northern part of Germany it was quite common, and they certainly had skilled physicians there ; he thought it might be due to arrest of development.

Dr. Post drew attention to the two classes of cases, one of which seemed to be rachitic ; but he had seen it occur in healthy infants with a difference in length of the limbs A few weeks since, he saw a case in which the head of the bone was in the thyroid foramen ; the speaker could only account for it that the child moved in utero.

Dr. Link, of Indianapolis, Indiana, read a paper on

ALCOHOL AS AN ANÆSTHETIC.

The speaker had used it in over a hundred cases and never had a fatal result, while the anæsthesia was complete. The whiskey to be given in two-ounce doses, every two to five minutes, until a pint to one and a half pints had been taken and the patient became stupefied. Then about two drachms of chloroform were placed in the cone, and a few respirations would put him to sleep ; the operation could then be carried out. The speaker's reasons for using this method was that in cases of shock there is depression ; the alcohol increases the heart's action, while the choloroform, which is a depressant, administered as stated, secures the equilibrium of the heart's action.

---

### SECTION ON MILITARY AND NAVAL SURGERY AND MEDICINE.

### Fifth Day—Morning Session.

AMPUTATION FOR INJURY OF LIVING PARTS NEVER NECESSARY.

A paper upon this subject was read by Professor Elisha H. Gregory, of St. Louis, Missouri. The author mainly described improved methods of coaptating deep structures, and the prevention of wound accidents and complications. He advises that, if by peculiarity of the injury the main channels are cut off, the surgeon should wait for collateral circulation, because the injury is not general.

The second paper was entitled,

THE SUPERIORITY OF THE BAVARIAN PLASTER-OF-PARIS SPLINTS FOR GUN-SHOT AND OTHER FRACTURES OF THE LIMBS, by James H. Gregory, of Omaha, Nebraska. The author related his experience and observation during four years of service as surgeon during the War of the Rebellion. Instances were given in which the spica-bandage and other appliances had caused gangrene.

The history of the Bavarian plaster-of-Paris splint was briefly given, after which he related his experience in its use, and the proper mode of its application.

This was followed by a paper by Richard Francis Tobin, of Netley, England, entitled,

SOME REMARKS UPON THE KIND OF DRESSING MOST AVAILABLE FOR GUN-SHOT FRACTURE OF THE LOWER LIMBS ON THE FIELD IN CONNECTION WITH TRANSPORTATION.

The paper, owing to the absence of its author, was read in abstract by Dr. Jeffrey A. Marston.

It had reference chiefly to fractures of the upper third of the femur, and to the desirability, in treating such cases, of fixing in an immovable position, not only the injured member, but also the opposite limb and the trunk. The appliances that, in the opinion of the writer, best fulfil these indications, were described.

Dr. J. R. Smith, U. S. Army, opened the discussion with the assertion that the permanent immobilization of fractured limbs upon the field of battle is impracticable.

The injured limb should be rendered immobile temporarily by a common splint, and by being fastened to the other limb, if the leg is involved, or to the body, if the arm be the seat of the injury. In the United States army all these modes and materials had been tried, and a large number of surgeons have about concluded that the silicate bandage is the neatest, lightest and strongest dressing for hospital use; the speaker had known patients thus treated to travel from Philadelphia to Maine.

Dr. R. B. Bontecou, of Troy, New York, preferred the silicate of soda or potassa as a primary dressing for fractures of the lower extremities, with a view to transportation; it is cheap, hardens quickly, is lighter than plaster-of-Paris, and can be carried in the field in tin cans of a capacity of one or two litres with a large mouth and screw-cap cover. When used it should be of the density of ordinary syrup.

---

### Afternoon Session.

The session was opened, and Dr. E. Griswold, of Sharon, Pennsylvania, read a paper entitled,

WHAT CONDITIONS ON THE FIELD JUSTIFY AMPUTATIONS IN GUN-SHOT WOUNDS?

The author claimed that amputation on the field of battle cannot always be done, although the established rule of surgery, as well as the opinion of the surguon, may demand it in many cases in which it is *not done.* The exigencies of war often separate the surgeon from his equipments and deprive him of the means of action, or he may be overworked and unable to do his work.

The surgery of to-day and of the future makes it necessary for military surgeons to be supplied with means by which asepsis can be practised upon the field in the treatment of wounds, and this can be done and made practical, wherever an adequate supply of water is attainable, if a rapid method of filtration and purification is available.

The possibilities of antiseptic surgery are already largely developed, and the military surgeon of the future must know what is to be expected in the way of conservatism, and when his patient, having a severe gun-shot wound in one of his extremities, is found to be clearly beyond the reach of conservatism, he is not only justified in amputating in the field, but it is his duty to do so.

Dr. Bontecou, Troy, New York, said he would practice conservative surgery when there are arteries to nourish the limb and veins to carry the blood back from the injured member to the heart.

Dr. E. A. Wood alluded to other elements, such as shock, which must be taken into consideration in determining whether amputation be or be not required, and in some instances of probable deformity resulting with consequent uselessness of the limb, the part should be excised.

Dr. Collins, of Philadelphia, Pennsylvania, thought many cases that would otherwise recover died of the wear and tear incident to the transportation.

Dr. H. H. Biedler, of Baltimore, Maryland, adverted to certain operations for grave injuries having terminated fatally after amputation, from the operation itself, and without the complication of shock.

Dr. Griswold closed the discussion by saying that he had noticed that shock, in injuries of the knee-joint, seemed to be more serious than in those of the hip joint.

When the injury is such that amputation is clearly indicated, it should be done early; still, even in bad cases, conservative surgery was available, and an attempt should always be made to save the limb.

A paper presented by Dr. Charles Smart, United States army, was, at the request of the author, read by title.

The President announced that the business before the Section was concluded, and with a few appropriate remarks declared the Section adjourned.

---

### SECTION ON OBSTETRICS.

### Fifth Day—Morning Session.

The committee appointed to formulate resolutions in regard to

UNIFORMITY IN OBSTETRICAL NOMENCLATURE, submitted its report, which, after discussion, was unanimously accepted, the only dissentient voice being that of Martin, of Berlin, who was not present but had left a message stating that he thought the matter should not be settled by the Ninth Congress, but should be deferred for three years and be accepted or not by a congress meeting in the Old World.

REPORT AS ACCEPTED.

A. It is desirable to try to attain uniformity in obstetrical nomenclature.

B. It is possible to arrive at uniformity of expression in regard to: 1, The Pelvic Diameters; 2, The Diameters of the Fœtal Head; 3, The Presentations of the Fœtus; 4, The Positions of the Fœtus; 5, The Stages of Labor; 6, The Factors of Labor.

C. The following definitions and designations are worthy of general adoption by obstetric teachers and authors:

I. Pelvic Brim Diameters.—1. Antero-posterior: (1) Between the middle of the sacral promontory and the point in the upper border of the symphysis pubis crossed by the *linea terminalis*=*Diameter Conjugata vera*, Cv. (2) Between the middle of the promontory of the sacrum and the lower border of the symphysis pubis=*Diameter Conjugata diagonalis*, Cd.

2. Transverse: Between the most distant points in the right and left ileo-pectineal lines=*Diameter Transversa*, T

3. First Oblique: Between right sacro-iliac synchondrosis and left pectineal eminence=*Diameter Diagonalis Dextra*, D. D.

4. Second Oblique: Between left sacro-iliac synchondrosis and right pectineal eminence=*Diameter Diagonalis Lœva*, D. L.

II. Fœtal Head Diameters.—1. From the tip of the ocipital bone to the centre of the lower margin of the chin=*Diameter Occipito-Mentalis*, O. M.

2. From the occipital protuberance to the root of the nose.=*Diameter Occipito-Frontalis*, O. F.

3. From the point of union of the neck and occiput to the centre of the anterior fontanelle=*Diameter sub-Occipito-Bregmatica*, s. O. B.

4. Between the two parietal protuberances=*Diameter Bi-Parietælis*, Bi-T.

5. Between the two lower extremities of theco-ronal suture=*Diameter Bi-Temporalis*, Bi-T.

III. PRESENTATION OR LIE OF THE FŒTUS.—The *presenting part* is the part that is touched by the finger through the vaginal canal, or which, during labor, is bounded by the girdle of resistance.

The *occiput* is the portion of the head lying behind the posterior fontanelle.

The *sinciput* is the portion of the head lying in front of the *bregma* (or anterior fontanelle).

The *vertex* is the portion of the head lying between the fontanelles and extending latterly to the parietal protuberances.

Three groups of presentations are to be recognized, two of which have the long axis of the fœtus in correspondence with the long axis of the uterus, while in the third the long axis of the fœtus is more oblique or transverse to the uterine axis.

1. Longitudinal: (1) Cephalic, including—vertex and its modifications; face and its modifications; (2) pelvic, including—breech; feet.

2. Transverse or trunk, including shoulder, or arm and other rarer presentations.

IV. POSITIONS OF THE FŒTUS.—The positions of the fœtus are best named topographically, according as the denominator looks—first to the left or the right side, and second, anteriorly or posteriorly. When initial letters are employed it is desirable to use the initials of the Latin words.

In the case of vertex positions we have:

Left Occipito-Anterior=*Occipito-Læva-Anterior*, O. L. A.

Left Occipito-Posterior=*Occipito-Læva-Posterior*, O. L. P.

Right Occipito-Posterior = *Occipito-Dextra-Posterior*, O. D. P.

Right Occipito-Anterior=*Occipito-Dextra-Anterior*, O. D. A.

The Face positions are:

Right Mento-Posterior = *Mento-Dextra-Posterior*, M, D. P.

Right Mento-Anterior = *Mento-Dextra-Anterior*, M. D. A.

Left Mento-Anterior = *Mento-Læva-Anterior*, M. L. A.

Left Mento-Posterior=*Mento-Læva-Posterior*, M. L. P.

The Pelvic positions are:

Left Sacro-Anterior=*Sacro-Læva-Anterior*, S. L. A.

Left Sacro-Posterior = *Sacro-Læva-Posterior*, S. L. P.

Right Sacro-Posterior = *Sacro-Dextra-Posterior*, S. D. P.

Right Sacro-Anterior=*Sacro-Dextra-Anterior*, S. D. A.

The Shoulder presentations are:

Left (left and right side of the *mother*) Scapula-Anterior=*Scapula-Læva-Anterior*, Sc. L. A.

Left Scapula-Posterior=*Scapula-Læva-Posterior*, Sc. L. P.

Right Scapula-Posterior=*Scapula-Dextra-Posterior*, Sc. D. P.

Right Scapula-Anterior=*Scapula-Dextra-Anterior*, Sc. D. A.

V. THE STAGES OF LABOR.—Labor is divisable into three stages: (1) First stage—from the commencement of regular pains till the complete dilatation of the os externum=*Stage of Effacement and Dilatation.* (2) Second stage—from dilatation of os externum till complete extrusion of child=*Stage of Expulsion.* (3) Third stage—from expulsion of child to complete extrusion of placenta and membranes=*Stage of the After-birth.*

VI. THE FACTORS OF LABOR ARE—(1) The Powers. (2) The Passages. (3) The Passengers.

(Signed) DELASKIE MILLER, M. D.,
*President of the Section.*
A. F. A. KING, M. D.
WILLIAM T. LUSK, M. D.
A. R. SIMPSON, M. D.

Dr. R. S. Stringer, of Florida, presented a paper on

A RATIONAL METHOD OF RELIEVING ASPHYXIA NEONATORUM, which, in the absence of the author, was read by title.

Dr. Ira E. Oatman, of San Francisco, California, read a paper on the

TREATMENT OF PUERPERAL ECLAMPSIA.

After giving a sketch of the disease and the usual methods of treatment, he recommended: When the convulsions occurred before labor, anæsthesia and clearance of bowels and rectum, together with as rapid delivery as was compatible with the safety of the patient. When the convulsions occurred after delivery and were accompanied by high nervous and vascular tension, nothing was so safe, speedy and reliable as veratrum viride. This he gave in the dose of ♏vj. by mouth, or ♏x. by rectum, and repeated it every fifteen minutes until the convulsions ceased.

Should the veratrum cause excessive depression an immediate and certain antidote was found in alcohol. It was better to give the veratrum in excessive doses than to risk the continuance of the convulsions; for the brandy or whisky to counteract its effects was always present, and the veratrum certainly exhibited a remedial action not otherwise attainable. The puerperal period was to be treated as usual, together with treatment of any pathological condition present. He found the veratrum equally efficient in the convulsions of children and in hystero-epilepsy.

Professor Alexander R. Simpson, of Edinburgh, Scotland, thought any suggestion that would enable us to control eclampsia of extreme value. He would like to hear opinions regarding veratrum; he had used it once, twenty years ago, and had not felt inclined to try it again.

Dr. G. Lane Taneyhill, of Baltimore, Maryland, advocated strongly the use of veratria in eclampsia, and said that fifty per centum of his cases had, in the last twenty years, been treated by it. He gave ten drops of the tincture hypodermatically every hour of convulsive action, and always kept another hypodermic syringe ready, filled with brandy to sustain the heart should the pulse go below forty-two. He was led to this practice by observing its use by Latimer in a case which he could control but temporarily by chloroform. He had not lost one case of eclampsia since using veratria; it enabled him to hold the engine, the heart, in check as an engineer with the air-brake, and was not as dangerous a medicine as was generally supposed. It produced the same effect as bleeding, save that no blood was lost to weaken the patient afterward.

Dr. Duncan C. MacCallum, of Montreal, Canada, had fourteen cases; all recovered. He used chloral, chloroform, potassium bromide, bleeding, morphia hypodermically; cleaned out the bowels by stimulating enemata. There was no routine treatment; each case was to be treated on its own merits.

Dr. Pierce, of Ohio, thought it unwise to try to map out any one line of treatment for eclampsia; each case must be rationally treated.

Dr. Lawrence, of Bristol, England, believed in as early delivery as possible, and the use of chloral. In plethora, bleeding was sometimes useful. It was difficult to lay down definite treatment.

Dr. G. W. Jones, of Danville, Illinois, said that veratrum is an active purgative, and a loaded colon is an active agent in causing epileptiform convulsions. He took charge of his patients beforehand, kept their bowels regular, used chloroform in labor when any nervous symptoms appeared, and saw no cases of eclampsia.

Professor A. F. A. King, of Washington, D. C., had never used veratria in eclampsia, but had suggested its use twenty years ago theoretically (*New York Medical Journal*, October, 1865), to lessen arterial hyperæmia of the nerve-centres, by reducing the force of the heart. He then, and more recently (in the *American Journal of Obstetrics*, March and April, 1887), considered, and still considers, eclampsia to be caused chiefly (and in addition to uræmic poisoning) by abnormal pressure of the gravid womb upon the aorta and its branches and vena cava and its branches. He believes this abnormal pressure to be chiefly due to the premature descent of the lower uterine segment and fœtal head *below the brim into the pelvic cavity*, especially in primiparæ two or three months before full term.

In transverse presentations, whether in multiparæ or primiparæ, eclampsia does not occur. The head of the child ought not to sink below the pelvic brim before labor—in fact, such *descent* is the *second* stage in the mechanism of *labor*. Yet recent authorities affirm that it is usual, and therefore normal, in primiparæ. This he denies. He maintains that the normal posture of the fœtus during pregnancy, before labor begins, is transverse or oblique; a head presentation is abnormal until the sinking of the womb just before delivery, when it changes in order that labor may take place. To remove the cause of and prevent eclampsia, we should, by posture and manipulation, lift the prematurely descended head out of the pelvis, and put it above the brim in one of the iliac fossæ, where it normally belongs, and where it would have remained but for corsets, dress, coitus and other displacing influences.

Professor W. W. Jaggard, of Chicago, Illinois, said: 1. Eclampsia is a symptom of renal insufficiency, renal inadequacy, to use a term of Sir Andrew Clarke's, the result of functional or organic disease. The arguments of Professor A. F. A. King were entirely inconclusive. 2. The cause of the convulsions, cerebral anæmia, the result of vaso-motor spasm, caused by irritation of the vaso-motor centre, by excrementitious matter retained in the blood. 3. Indication for treatment, absolute, continued narcosis. Chloroform, potassium bromide, chloral, morphine. Veratrum viride, according to researches of Wood and Behrens, of Philadelphia, causes vaso-motor dilatation, and the woman is literally "bled into her own veins." At the same time, when this remedy is employed a hypodermic syringe filled with brandy must be ready. Pilocarpin is unreliable, causing cardiac depression, and aiding in

œdema pulmonum when no diaphoretic action is effected. Professor Fordyce Barker has frequently called attention to this fact.

Dr. Oatman said that he had seen doses of as much as a drachm of the fluid extract of veratrum taken by mistake, and recovery, without treatment, had occurred.

---

AFTERNOON SESSION.

Dr. H. O. Marcy, of Boston, Massachusetts, read a paper on the

HISTOLOGY AND PATHOLOGY OF REPRODUCTION.

The studies offered were based upon the conviction that the profession owes to the late Professor Ercolani, of Bologna, the establishment of new and simple truths which are fundamental and of the first importance. Comparative studies teach that there is a unity of type in placental development. The writer endeavored to show that immediately after conception a destructive process affects the inner surface of the uterus; in some of the lower animals and in women this process is limited to the epithelium, while in other animals, as in the rodents, the destruction extends to the entire sub-mucous connective-tissue layer.

This destruction is essential, since it facilitates the setting up of neo-formative changes, from which will result the maternal portion of the placenta. This process consists in the formation of new vessels, which are distinguished from the vessels of the unimpregnated uterus in that both the artery and vein consist of only a simple endothelial wall, and that from the external surface of this is elaborated a layer of special cells not separable from the wall of the vessel. These are the so-called decidual or placental cells.

The relation established between those two factors of new formation is what is known as placental development. The manner in which this relation is established gives rise to the different forms of the mammalian placenta.

Professor Alexander R. Simpson, of Edinburgh, Scotland, spoke of the value of the paper and sketched the development of the placenta from the lowest type, where the fœtal and maternal surfaces were simply laid together, and were separated at parturition, to the complicated interdigitation in the higher animals, and the highest type as found in the female, where, at the time of delivery, the maternal as well as fœtal portions came away together, leaving beneath it only the basal portion of the uterine mucous membrane.

Dr. E. Paul Sale, of Aberdeen, Mississippi, read a paper on the

MANAGEMENT OF PREGNANCY, WITH REFERENCE TO THE PREVENTION OF POST-PARTUM HEMORRHAGE, in which he strongly insisted upon the value of prophylaxis, undertaken months before delivery, during which time the patient should be under the supervision of the accoucheur. Some of the predisposing causes and their treatment are as follows:

1. Hæmophilia, abnormal tenuity of the walls of the capillaries. As treatment, use stimulating oils, such as turpentine or ol. origanum. Matico, liquid extract, is also good. Ergot and allied drugs have no value.

2. Anæmia, from various causes. For this use fluid extract stylothansis, cimicifuga racemosa, salix nigra, etc. Faradization of the abdominal muscles for ten minutes daily, is beneficial.

3. Atonic debility from rapid child-bearing. Exercise, tonics, quinine, strychnia, iron, fresh air, constitute the best remedy.

4. Laceration of cervix, cancer, fibroma. This is to be let alone, except before the third month.

5. Intemperance. Moral measures (what success?).

6. Heart disease. In this gelseminum is often useful.

7. Plethora. Dietetics and salines are best.

Dr. H. B. Hemenway, of Kalamazoo, Michigan, had not been able to diagnose post-partum hemorrhage many months before labor. He asked, what was the relation of late ligature of the cord to post-partum hemorrhage?

He strongly suspected that it tended to prevent such an occurrence, and his explanation was as follows: There is no direct connection between the maternal blood-vessels and the placenta; if the cord is ligated late, or if only the fœtal portion was ligated, blood flows from the placenta. The cord is normally attached near the center of the placenta; if, then, blood is extracted, the placenta must shrink and the edge will first become detached. If blood is not extracted from the placenta, it is more rigid, and as the uterus contracts the center is likely to be first detached. A sac is thus formed which fills with the maternal blood, and the mouths of the uterine vessels do not firmly close. When the placenta is delivered this accumulated blood rushes out and the maternal vessels again bleed.

Professor W. W. Jaggard, of Chicago, Illinois, called attention to the following propositions:

1. Late ligature of the cord is always indicated in the interest of the child—when not contra-indicated in the concrete case. The researches of Budin have demonstrated that when the cord is ligatured when it ceases to pulsate the child receives seventy-five grammes of blood —lost, when the pulsating cord is tied. The relation of late ligature of the cord to the prevention of post-partum hemorrhage, to which the gentleman from Michigan has called attention, is of great interest, if it be a fact.

2. The best preventive of post-partum hemorrhage is to secure retraction of the uterus, by keeping the hand on the fundus uteri from the moment the child begins to pass through the vulvar outlet until the muscular fibres have rearranged themselves—about one hour after expulsion of the placenta.

Kucher, in his admirable book, has called attention to friction over the fundus twice daily during the first two days of the puerperium. This is the practice in Vienna. There is little danger of dislodgement of a clot in the uterine sinuses, or interruption of the process of puerperal thrombosis, by this procedure. It is also an excellent prophylactic against resorption of septic matter.

Dr. George Wheeler Jones, of Danville, Illinois, read a paper on

DISTOCIA FROM RIGIDITY OF THE CERVIX AND ITS MANAGEMENT.

After a consideration of the various conditions which might cause rigidity of the cervix, he spoke of the most important—spasmodic contraction. Here opium was most valuable, together with chloroform and Barnes' dilators, if quick dilation was necessary. Sitz baths, warm vaginal douches, delivery in a warm room, morphia hypodermically, quinine where there was malaria, salicine in rheumatic cases, and electricity were all useful. He would never use chloral, as it was dangerous to mother and child. He then detailed some original investigations into the medicinal action of Gossypii radix, delphinium, and ipecacuanha.

Professor A. F. A. King, of Washington, D. C., called attention to the fact that the thinning of the uterine segment and the obliteration of the cervical canal, conditions which led to the rigidity of the cervix, were abnormalities. When the uterus and child maintained during pregnancy their normal lateral obliquity, the canal of the cervix from the external to the internal so will remain unobliterated until full term, which is the normal condition both for primiparæ and multiparæ. Under the same normal circumstances the great thinning of the lower uterine segment, the tearing of the decidual mucous membrane, the "formation of a new cervical canal," and the other tissue-changes observed by Brandl will be absent *during pregnancy.* They are abnormal. They may, nevertheless, occur *during labor* from *abnormal* mechanical obstruction to delivery. Professor King also objected to the use of chloral.

Dr. Brooks H. Wells, of New York, N. Y., did not agree with Dr. Jones concerning the great danger to the unborn child from the use of chloral. In about one hundred cases out of between six and seven hundred which he had had under his charge, or had witnessed, it had been used both as an anæsthetic, where the first stage had been unusually painful, and in spasmodic rigidity of the cervix, and in no case had he seen any harm accrue to mother or child, but only the most gratifying results. He always used the precautionary measure mentioned by Dr. Jaggard, viz., to keep himself informed of the condition of the uterus by a hand placed over the fundus, slight rotary friction overcoming any possible tendency to relaxation, and had never had any serious post-partum bleeding, either with or without the use of chloral. Its administration might, in full doses, blunt the pains somewhat. He was accustomed to administer the drug in ten to fifteen grain doses, by mouth or rectum, until this result was attained—usually from two to four doses. He considered morphia and hot douche important agents in treating the class of cases mentioned.

A paper by Dr. John H. Wilson, of Chicago, Illinois, entitled "Puerperal Uræmic Amaurosis," was read by title.

The Section then adjourned.

---

### SECTION ON GYNÆCOLOGY.

### Fifth Day—Morning Session.

Dr. Henry O. Marcy, the President of the Section read a paper on

THE HISTOLOGY AND SURGICAL TREATMENT OF UTERINE MYOMA.

The author presented an interesting array of microphotographs. He showed the multilocular growth; the capsule; the multilobular growth and the parts at which they lie in juxtaposition; the histological relation of the growth to the uterus; the vascularization; the first injection they were able to make into a fibroid tumor; and discussed the bacilli discovered by Dr. Nelson, of Boston, of which he termed bacillus Nelsonii. He showed a number of other cuts, and the specimen of a tumor which he had removed from a woman per vaginam, but which he would not attempt again, on account of its size; he exhibited some instruments for the operation of removal of fibroid tumors, of his invention, and discussed the shoemaker's stitch.

Illustrations were thrown on the screen by Drs. Martin, Clark, Nelson and Hewson.

Dr. Caleb R. Reed, of Middleport, Ohio, read a paper on

INTER-UTERINE STEM PESSARIES, in which he favored very much their use for promoting the flow of the menses in cases of local origin, and especially those where there was an ill-developed uterus. He thought that this instrument should come in the list of emmenagogues, and that it should not be condemned with the arrogance which sometimes accompanies its discussion.

Dr. Daniel T. Nelson, of Chicago, Illinois, read a paper on

THE TREATMENT OF UTERINE MYOMA BY ERGOT, and gave the statistics which he had collected from the profession and made a good argument for this mode of treatment.

A paper by M. D. Spanton, M. D., of Hanley, England, was read by Dr. Edoes, of Manchester, England, on the subject of

CYSTITIS IN WOMAN.

Local conditions leading to cystitis in women, among which are enumerated diseased states of the uterine appendages, bands of adhesions dragging upon the bladder, and some of the affections causing obstruction to the passages of the uretha, which are often obscure and trivial in themselves and not infrequently overlooked.

Dr. William L. Reid, of Glasgow, Scotland, read a paper on

THE REMOTE RESULTS OF SHORTENING THE ROUND LIGAMENTS.

He had performed the operation eighteen times. He gave the opinions of authorities, mostly British, and which were mostly unfavorable to the operation. The discussion on this paper was also rather unfavorable to the operation.

---

AFTERNOON SESSION.

Dr. J. H. Kellogg, of Battle Creek, Michigan, read a paper on

ALEXANDER'S OPERATION, and reported twenty cases. He favored the operation.

Dr. W. C. Wade, of Holly, Michigan, read a paper on

DISPLACEMENTS OF THE UTERUS.

His paper consisted of thirty-nine terse statements about displacements.

Dr. Joseph Tabor Johnson, of Washington, D. C., read a paper on

THE TREATMENT OF COMMENCING OR THREATENED PERITONITIS BY BRISK PURGATION.

Professor Vulliett, of Geneva, Switzerland, had two papers, *Progressive Uterine Dilatation and the Buried Suture with Iodized Silk in Vesicovaginal Fistula.* They were read by Professor Cordes, of Geneva.

Dr. Addinell Hewson, of Philadelphia, Pennsylvania, read a paper on

ABDOMINAL SURGERY.

Dr. E. H. Trenholme, of Montreal, Canada, read a paper on "Extirpation of the Uterus for Bleeding Myoma."

A number of papers were read by title and the Section adjourned.

---

SECTION ON THERAPEUTICS AND MATERIA MEDICA.

FIFTH DAY.

The following papers were read by title: "On Collinsonia Canadenisis," by John V. Shoemaker, M. D., of Philadelphia, Pennsylvania; "On Cold as a Remedy in Inflammatory Affections," by Hiram Corson, M. D., of Conshohocken, Pennsylvania; "The Therapeutics of Alcohol in Disease," by E. N. Liell, M. D., of New York, N. Y.

Dr. Justus Andeer, of Munich, Bavaria, read a paper on

RESORCIN AND ITS PREPARATIONS.

In this paper the author briefly gave the result of ten years' experience with pure resorcin. He had found it non-toxic, antiseptic, and possessing many advantages over carbolic acid, being free from odor and non-corrosive to the skin. He had obtained excellent results from it in ezema, in keloid and in parasitic skin diseases, as well as diseases of mucous membranes. In the form of keratine-coated pills, it can be given for bronchitis, or in combination with castor-oil (one to two per cent.) for diarrhœa and dysentery. For local use it may be combined with cacao-butter as a salve, or it may be made up into a soap of varying strength, from one per cent. up. Failure to obtain satisfactory results from its use may arise from not using a chemically pure resorcin, or from giving it in insufficient doses.

Dr. Lewin, of Berlin, Prussia, said that all are familiar with the writings of the author upon the subject of resorcin. He thought that by devoting exclusive attention to this one member of a group the others might be in danger of having their value slighted. What is the value of thymol? What does resorcin accomplish more than phenol, carbolic acid, and others of this class? He questioned the statement that resorcin is not toxic, since it can in large doses present a condition of cerebral confusion and excitement which closely resembles mania.

Dr. Andeer replied that the agent was an efficient antiseptic, tasteless, odorless, and in ordinary doses free from toxic effects. He considered it a valuable addition to our therapeutic resources, and superior to phenol and thymol in many respects.

Dr. William Ward, of Washington, D. C., presented a paper on

VICARIOUS RESPIRATION SUPERINDUCED IN CERTAIN DISEASES BY ADMINISTRATION OF OXYGEN-YIELDING COMPOUNDS THROUGH THE ALIMENTARY TRACT.

The reading of this paper was not finished when the hour of adjournment arrived.

---

### SECTION ON ANATOMY.

### Fifth Day—Morning Session.

Dr. J. Neely Rhoads, of Philadelphia, Pennsylvania, read a paper on

THE ANATOMY OF STRICTURE, in which he reviewed the anatomy of the urethra, and, after comparing the male and female urethra, laid it down as a fact that the former was not a canal of uniform calibre, while the latter was. He also dwelt upon the great sensitiveness of the meatus in both sexes. After describing the different varieties of stricture, he exhibited a full set of pocket bougies, as made by Snowden, of Philadelphia. This case contained ten 2¾-bulbs, a universal handle, and several different sound and bougie points, making a cheap, easily portable, and convenient case, equivalent, by varied combination, to twenty bougies. The principal advantage claimed by him was that the bulbous part, being in the centre of the sound, the meatus was only dilated up to a certain point, and then the smaller handle prevented further pain, while the bulb, deeper in the urethra, was dilating the stricture.

Dr. E. C. Spitzka, of New York, N. Y., then read a paper on

THE MESOCEPHALON OF THE TRUE REPTILE, and one on

THE INTER-CRANIAL NERVE-TRACTS IN THE LIGHT OF ATROPHY-METHODS OF VON GUDDEN.

The observations related to two subjects which were somewhat connected. The first has the significance of the fact that the post-optic lobes varied so much in the animal scale as to have actually been overlooked in some reptiles and amphibia, and to be enormously overgrown in others. He believed to have found this in the relation of these bodies to the auditory nerves. Where these were large the post-optic lobes were large. This observation was confirmatory of the experiments of Baginsky and others.

In the discussion which followed Dr. Frank Baker, of Washington, D. C., asked Dr. Spitzka if he could trace the nerve-tract throughout its whole course. He remarked on the importance of the conclusions and asked as to the course of the atrophied nerve-tracts. The metameric division of the brain and skull could not, he thought, in the present condition of our embryological knowledge, be clearly made out. There could be no doubt that the number of somites was large, and as the differentiation had been very great it was doubtful whether we could properly determine the segmental value of each nerve. The auditory nerve was probably complex. In conclusion, he asked what Dr. Spitzka had observed as to the separate origin of the cochlear and vestibular branches.

Dr. Augustus C. Bernays, of St. Louis, Missouri, in reply to Dr. Spitzka, remarked that the sense of hearing was only a modification of the sense of touch, and that the auditory nerve was probably only a sensory portion of the same spinal nerve of which the facial was the motor portion. He could not see how the nerve of hearing could become attached to the post-optic lobes. He thought that the theory of Dr. Spitzka could not readily be made to agree with this proposition.

Dr. Spitzka, in reply to Dr. Bernays, said that while recognizing the importance of the segmental theory, he did not think it advantageous to engraft biological cranial nerve-anatomy on physiological cranial nerve-anatomy. If the two sets of observations conflicted, he would sacrifice the former.

In answer to Dr. Baker he replied that the difference between the two divisions was recognized; the vestibular branch went to the cerebellum. His observation related only to the cochlear branch.

Dr. A. H. P. Leuf, of Philadelphia, Pennsylvania, then read a paper on

THE PROPER METHODS IN THE STUDY OF ANATOMY, in which he enumerated the many defects in the present mode of teaching anatomy, and, confining his remarks principally to schools in this country, he denounced all schools, with few exceptions, in this respect as bad and unscientific. His list of defects is as follows: (1) The instruction is special as regards certain organs only. (2) It is too general as regards other organs. (3) It is neither the one nor the other as regards still other parts of the body. (4) In generally overlooking the relations of organs to one another in a way that would be very useful to remember. (5) Failure to prepare the student's mind with an incentive to acquire anatomical knowledge before it is imparted. (6) Too little of the philosophy or principles of anatomy, which, if properly taught, would make remembrance easier. (7) The failure to clearly show the connections of anatomy with the other branches of medicine. (8) The great want of good teachers.

To remedy these many defects, he thought it necessary: (1) To teach the special anatomy of *every* organ, distinct from all others. (2) To also give a general idea or outline of each organ. (3) To never fail to do this with every organ or part of the body. (4) To invariably show the true and exact relations of the parts to each other. (5) To always create in the mind of the student a desire for the knowledge to be acquired, by showing its necessity. (6) To show in a natural way, without much artificial mnemonics, how many anatomical principles there are which, if remembered, will do alike for all parts of the body. (7) Thorough instruction as to the relations of anatomical study to the other branches of medicine. (8) To have good teachers and men thoroughly acquainted with the subject.

In the discussion which resulted Dr. Augustus C. Bernays, of St. Louis, Missouri, objected strongly to the use of mnemonics or any other artificial means of learning anatomy, and believed that it should be taught from an embryological stand-point alone.

Dr. Frank Baker, of Washington, D. C., was much opposed to the manner of teaching anatomy where only a collection of isolated facts was given. His definition of anatomy was the science of structure.

Dr. E. C. Spitzka, of New York, spoke in strong terms against the pernicious methods of teaching anatomy, and thought we should try to raise the standards of our study of anatomy to a level with those in Europe.

Dr. F. W. Langdon, of Cincinnati, Ohio, then read by title a paper on

THE DIDATIC TEACHING OF HUMAN ANATOMY.

---

AFTERNOON SESSION.

The afternoon session was of short duration, and much time was occupied by Drs. F. C. Shaefer, Joseph N. Dickson, and others in discussing the subject of

SKIN GRAFTING.

The President, Professor Pancoast, of Philadelphia, gave a demonstration of the peritoneum and its position in relation to the abdominal organs, by means of different-sized sponges and mosqueto-netting. This demonstration is of great assistance in making clear to students the manner in which the peritoneum, a closed sac, surrounds the abdominal viscera. He also stated his theory as to the development of the peritoneum. The Section then adjourned.

---

### SECTION ON DISEASES OF CHILDREN.

SEPTEMBER 9TH—FIFTH DAY.

Dr. A. B. Judson, of New York, N. Y., Vice-President of the Section, called the meeting to order. He said that it might be called the first meeting of the Subsection of Orthopædic Surgery, the embryo of what may in the future be a mature section.

A paper was read by Mr. William E. Balkwill, of London, entitled

THE TREATMENT OF CONGENITAL CLUB-FOOT, DEMONSTRATED BY THE EXHIBITION OF APPARATUS.

The writer limited himself to the consideration of talipes equino-varus. He believed that in the majority of cases tenotomy will be required. The question of an early or late operation is to be answered by considering the general condition of the child rather than the nature of the deformity, as a severe case may be as thoroughly and permanently reduced, if circumstances make a delay desirable, a few months after birth as if the operation was done in the first week. He divides the tibialis posticus, the tibialis anticus, and the plantar fascia, in the order named, and after an interval of three weeks of the diligent use of a flexible splint and bandages, he is ready to proceed with the division of the tendo Achillis. Then, instead of extending fully and at once after tenotomy of the Achillis, and keeping the foot in the new position, he takes more time, and, in fact, uses the foot as Isaac Walton did his frog—no force, no undue pressure, but careful guidance.

Mr. Balkwill then exhibited a large number of ingenious instruments designed to accomplish complete reduction by the gradual method. Their action was controlled by the pinion-key in some instances, and in others by modifying with suitable wrenches the tractable steel of which they were constructed. He deplored the existence of a strong prejudice among people of all classes against the wearing of "irons." If properly constructed and correctly applied, they should do the work required, not only without pain, but with an increase of comfort, arising from the increased facility which they add to locomotion. He feared that surgeons were largely responsible for the existence of this prejudice, from the fact that it is so mnch easier to refer the case to an instrument-maker than to give to it the care and painstaking which it requires and without which success is impossible.

A paper was then read by Professor Lewis A. Sayre, New York, N. Y., entitled

SECTION OF CONTRACTURED TISSUES ESSENTIAL BEFORE MECHANICAL TREATMENT CAN BE EFFECTUAL.

The writer defined his position as follows: A contrac*ted* tissue is one that is simply shortened, but which can be elongated by careful, continuous, and judiciously applied traction and manipulation. A contrac*tured* tissue is one which has undergone some change of structure in the fibrillæ of the muscles, and which cannot be elongated or stretched. Distortion or deformities which are the result of contrac*tured* tissue can, only be removed by forcible rupture of the same, or by cutting before traction is applied; but similar deformities resulting from simply contracted tissue may frequently be rectified by manipulation and constant traction, properly applied, without section of the tissue. How are we to know whether the tissue is *contracted* or *contractured?* If in any case of club-foot or other deformity from muscular contraction we stretch the shortened parts to their utmost tension by manual force or mechanical aids, and when the parts are thus stretched we suddenly add to the tension by pressing with the thumb or finger on the part thus stretched, or by pinching the stretched tissue between the thumb and finger, and if by either of these acts we produce a *reflex* spasm, or sudden shivering of the whole body, that muscle, tendon, or tissue, thus yielding this *reflex spasm* is *contractured*, and cannot be elongated *without severing* of its fibres. If, on the contrary, when the test is applied as above described, and no *reflex* irritation or muscular spasm is produced, it is evident that the parts are simply *contracted*, and can be further elongated by persistent constant traction and proper manipulation, and therefore do not require divulsion.

To attempt to stretch a contractured tissue causes unnecessary pain, and exposes the patient to the risk of disturbances of the nervous system through long-continued irritation. In all such cases it is infinitely safer to make such division by subcutaneous section than by manual or mechanical force. Treatment is to be completed by massage, friction, active and passive motion, electricity, and such mechanical appliances as may be required in each case. A number of illustrative cases were given.

The Vice-president, Dr. A. B. Judson, thanked Mr. Balkwill for bringing over the instruments which he had shown, braving the terrors of the New York Custom House in order to add to the interest of the Congress. His view of the subject was that the treatment of congenital club-foot is divided into two stages by the arrival of the time when the child begins to try to stand. Before this time, when the child's weight does not interfere with corrective procedures, the varus should be reduced by whatever means may be at hand—a succession of carved wood splints, tractable metal splints, adhesive plaster, plaster-of-paris splints, the artificial muscles; or a combination of these methods, one with another, or with tenotomy, as the case may require. Whatever the plan adopted, by the time the child begins to put his weight on the foot, the varus should be so far reduced that the sole is presented squarely to the ground. Of course, if nothing more be done, the varus would return when walking begins, and would soon be as bad as ever; and what is now required in this, the second stage, is a slight splint extending up the inner side of the foot and leg, and so fitted with buckles and webbing that pressure may be distributed where the steel is directly applied, and concentrated on the outer side of the ankle, where it can be easily borne, because made by webbing-straps. The object of the splint is simply to give the foot a push toward valgus, so so that when the child steps the sole presents squarely to the ground, or a little more than squarely. Every foot-fall will then be an impulse in the right direction, applied with the force of the rapidily increasing weight of the body. With tractable metal in the splint varus may be absolutely reduced, and, if it were desirable, even valgus could be produced.

Mr. Edmund Owen, of London, England, stated that he was heartily devoted to what he believed was rightly called the American plan. The old plan consisted in dividing first the tendon of the tibialis posticus, and perhaps also that of the flexor longus digitorum, and then in putting the foot up in only a slightly improved position; then, when it was supposed that plastic effusion had connected the divided ends of the tendon, to begin with Scarpa's shoe to correct the inversion. Later on, perhaps weeks afterward, when the inversion had been fairly dealt with, the tendon of Achilles was divided, and in a few days flexion of the foot was started with. Mr. Owen considered that this plan was now, even in England, giving place to the method of dividing all the tendons at one and the same time; and not only that, but bands of fascia and ligament were also divided. He called particular attention to the need of dividing the anterior part of the internal lateral ligament of the ankle-joint, which was much concerned in keeping the frontpart of the tarsus flexed and inverted on the head of the astragalus. Scarpa's shoe should, together with mechanical spinal supports, be relegated to the limbo of extinct surgical instruments; the introduction of the plaster-of-Paris treatment had rendered them superfluous. There need be no fear of failure of union occurring in the divided tendon after putting the foot at once in the correct position; plastic effusion and blood in the sheath of the tendon quickly prepared the way for an efficient splicing of the tendon.

Mr. Owen could not accept entirely all that Professor Sayre said as to the difference between contractured and contracted tendons ; he would not deny its existence in fact, but he was not prepared entirely to accept it. He commended, in the highest terms, Dr. Sayre's teaching and his practice.

Dr. E. R. Lewis, of Kansas City, Missouri, said that if the distinction made by Professor Sayre was correct, it was a most valuable diagnostic sign. He would try it, and he hoped to be successful.

Dr. Hingston, of Montreal, Canada, stated that formerly it had been his custom to delay tenotomy till the period of teething, at least; but he had long ago accepted the dictum of Dr. Sayre, that one is warranted in delaying the operation only till the tenotome can be procured. He had followed this rule with almost invariable satisfaction.

Professor D. A. K. Steele, of Chicago, Illinois, advocated the plan of treatment which had been outlined by Mr. Owen. His experience had led him to the adoption of the following rules of practice: (1) Operate as early as the child is brought. (2) Divide all tissues that prevent perfect restoration of the foot. (3) Retain the foot in a correct position by plaster-of Paris dressing. (4) Leave the toes out in order to watch the circulation. Antisepsis, of course to be secured.

A tripod was then erected, and Dr. Lewis A. Sayre and Dr. Lewis Hall Sayre suspended a patient of Dr. Magruder, of Virginia, and applied

THE PLASTER-OF-PARIS DRESSING FOR POTT'S DISEASE.

The following papers were afterwards read by title: "Forcible Correction of Contracted Knee-joint," by Dr. E. H. Bradford, of Boston, Massachusetts ; "The Progress of Orthopedic Surgery," by Mr. Noble Smith, of London, England; "Anchylosis of the Knee-joint in a Straight Position by Exoision, as a Remedy for the Atrophy and Deformity following Acute Poliomyelitis of Childhood," by Dr. Stephen Smith, of New York, N. Y.; "The Treatment of Lateral Curvature of the Spine," by Dr. James Knight, of New York, N. Y.; "The Results of the Treatment of Hip Disease by the so-called American Method," by Dr. A. B. Judson, of New York, N. Y.; "The Diseases of Faulty Habit," by Dr. James F. Goodhart, of London, England; "The Treatment of Strumous Glandular Enlargement of the Neck," by Dr. Martin G. B. Oxley, of Liverpool, England; "The Nature and Antiseptic Treatment of Whooping-cough," by Dr. Moncorvo, of Rio de Janeiro, Brazil; "On Acetonuria in Children," by Dr. Adolph Baginsky, of Berlin, Germany; and "Mediastinal Tumors in Children," by Dr. Oscar Wyss, of Geneva, Switzerland.

The Session was closed by a few valedictory remarks by the President.

At the close of the Session, initiatory steps were taken to form

AN AMERICAN PEDIATRIC ASSOCIATION, with Dr. J. Lewis Smith, of New York, N. Y., and Dr. W. D. Booker, of Baltimore, Maryland, Provisional President and Secretary.

---

### SECTION ON OPHTHALMOLOGY.

### Fifth Day—Morning Session.

The Section being called to order, Mr. Henry Power, London, England, presented a new pattern of *ophthalmoscope, folding handle*, for vest-pocket use, which could be carried in a case much like a pair of eyeglasses. A paper was also mentioned as having been sent by Dr. Prince, of Jacksonville, Illinois, *On Some Points in the Treatment of Dacryocystitis and Affections of the Nasal Duct*, with a new canula for drainage, which instrument was exhibited to the Section.

Professor E. Smith, of Detroit, Michigan, then read a paper on

THE TREATMENT OF ABSCESSES AND ULCERATIONS OF THE CORNEA WITH JEQUIRITY.

He remarked upon the experience, common to all, of the difficulty in procuring the absorption of pus in the cornea, and noted the existence of the same tendency in all closed abscesses. The best means of arresting suppuration and of getting rid of the pus when formed, has engaged the attention of the writer, with many others, for years. A paper on the above subject, in the spring of 1883, with another published in October, 1883, gave the result of his observations up to that time. He had since had many cases of ulceration of the cornea speedily relieved, and an astonishing clearing up of the cornea after its use. He believes the remedy affects the proliferation in corneal corpuscles. After explaining his impression of the mode of action of the remedy, he proceeded to describe his manner of using it. He does not use a strong preparation, and aims not to produce a sharp inflammation, as is done in trachoma. He uses a three per centum solution, or a very minute quantity of the powdered seed. It is applied sparingly, till a mild catarrhal inflammation is set up, characteristic of the remedy, and in some cases there may be slight membrane. The object is to avoid a high degree of reaction. The eye is kept washed out with a two per centum solution of boric acid, and the result is almost uniformly surprisingly good. The corneal cicatrix is often hardly apparent to anyone but the patient.

Dr. Galezowski, of Paris, France, did not like jequirity. In his observation it had often produced ulceration and destruction of the cornea and synechia anterior, and in some cases enucleation had to be performed to avoid sympathetic trouble. He thought jequirity exerted a very bad action on the cornea. The modification in Professor Smith's method is doubtless in the amount of the drug used. Dr. Galezowski thought it a dangerous method.

In intermittent fever he had found ulceration of the cornea, which yielded to the administration of quinine. In another case, lasting for several months, after the extraction of a tooth, cicatrization occurred. The best treatment for corneal abscess and ulcer is the antiseptic method. He applies the powdered iodoform directly to the surface three times a day, under cocaine, and uses the steam douche two or three times a day, ten minutes at a time. When it is not doing well cauterization with a solution of nitrate of silver, twenty-five centigrammes to ten grammes may be done once or twice a day.

Dr. S. O. Richey, of Washington, D. C., wished to know whether scraping the ulcer before applying any remedy had not been found of advantage by others as it had in his hands.

Professor Smith, in closing, said that Dr. Galezowski had used jequirity in trachoma, in the way described by De Wecker, till he obtained marked diptheritic membrane. In these cases it was the swelling and chemosis that caused strangulation and loss of cornea. He should hesitate to use jequirity in sthenic cases; it was the asthenic cases requiring stimulation that were suitable for this treatment.

Professor D. S. Reynolds, of Louisville, Kentucky, read the next paper on the

NECESSITY FOR REFORM IN THE MANNER OF DESIGNATING LENSES.

He said that often the difficulty was more in the defective apparatus than in the methods employed. The method which he employed was to abandon the idea of measuring and denoting lenses by their focal distance, and make the radius of curvature the measure of the lens, having them measured from a unit of ninety degrees, or the quadrant of a circle. The lenses might be graded at intervals of five or ten minutes, and in the higher grades have an interval of fifteen minutes. He gave a description of an instrument of Professor Snellen's for determining the value of a lens. It consists of a stand with upright, on which a horizontal beam carries at one end a lamp, and near it a positive lens of one-sixth. In the centre of the beam is placed a clamp for holding the lens to be tested. On each side of the centre, on the beam, is placed a lens plano-convex one-twelfth. Near the lamp is placed a copper disk, perforated by two lines of minute holes, one line perpendicular and the other horizontal. At the farther end of the beam, working in a graduated brass plate, is a frosted glass disk. Steel tapes pass up through the stand and along the beam, connecting with the frosted disk and the perforated plate. It is easy, by this means, to note the exact point where the point of light reaches the frosted disk without any red or colored margin, and by revolving the lens on trial in the clamp any irregularity can be easily ascertained.

Dr. Landolt, of Paris, France, wished to know how the essayist would express in writing the power of the glass by his proposed method. In reply, Professor Reynolds wrote the prescription for a supposed case:

O. D. | O. S. | Bis C.

$+\frac{1}{20}$c.90° | ⊃$+\frac{1}{40}$s. | $+\frac{1}{60}$c.75° | ⊃$+\frac{1}{60}$s. | 2½''

Mr. J. Richardson Cross, of Bristol, England, wanted to know the focal length of the unit of Professor Reynolds' system, and received the reply that it would be something less than an inch.

Dr. E. Landolt suggested that the radius of curvature, while most accurate as a unit of measurement, is not satisfactory, as with the same radius there may be differences in composition and differences in the refractive power. The manufacturers of lenses will tell you that the refracting index of lenses is a variable quantity, and no one can make lenses, even of the same material, that will always have an exactly similar refracting index.

Professor S. M. Burnett, of Washington, D. C., thought we could not get along without a knowledge of the focal distance of the lens. His objections to the proposed change were that Professor Reynolds' plan lacks simplicity, and that the present method seems to answer the purpose very well and is universally adopted, and that the change would be very difficult to make.

Mr. Cross thought that the proposed plan was based on scientific accuracy. But, as a reformer, the author must not expect to succeed until he can give us a medium which is as accurate as his quadrant. The speaker himself was perfectly content with the accuracy of the meter. Of course it is not quite accurate, but much more so than any other measurement we have. Then all refraction deals with focal length. Our patients want to see at a certain distance. We fit them with glasses to see to a particular point. Again, contrast the simplicity of the meter with the complicated formula of Dr. Reynolds. It is as bad af going back to the inch system.

Dr. G. W. Allyn, of Pittsburg, Pennsylvania, was unwilling to return to the old system of numerals in refraction. We deal constantly with distance, and distance is measured on a straight line. He, too, thought the adoption of the method would be a step backward.

Dr. J. L. Thompson, of Indianapolis, Indiana, thought we could not always make scientific methods practicable. He was well enough satisfied to write in the metric system. There was no certainty in the composition of spectacle lenses, even in pebbles, which are so much talked about all over the country, and of which so few are sold.

Dr. E. Jackson, of Philadelphia, Pennsylvania, then read a paper on

THE DESIGNATION OF PRISMS BY THE MINIMUM DEVIATION, INSTEAD OF BY THE REFRACTION ANGLE.

The object of the paper was to urge the advisability of extending the same principle to the numbering of prisms that now obtains in relation to lenses. The refracting power of the prism depends upon the substance of which it is composed, and upon the medium in which it is placed. The latter, being air, can be eliminated in practice. The same difference existing in the material of which prisms are made as in that of spherical and other lenses, it follows that prisms cut to the same angle may differ very much in the extent to which the ray is bent in passing through them. For instance, a prism marked 3° may have a refracting power of 1.51° or 2¼°, and yet be the correct one according to the prescription, by our present method of measument. Among a number of prisms tested, only one set came within sixteen per cent. of the supposed standard. He proposed to take as the basis of measurement the minimum deviation or the deflection of the ray when it passed though parallel in the prism to the base, or where the angles made with the surface on entering and leaving were the same. The change could easily be made, without producing confusion with the present system, by marking the number of the new system inside a circle, thus: (2).

Dr. E. Landolt, of Paris, expressed himself as much interested in the paper. It was exceedingly meritorious, logical and practical, and he would regard it as an honor to carry out the proposal to support the idea. He suggested that the section take up and support the opinion expressed in the paper.

Mr. H. Power, of London, England, moved that a committee be appointed by the chair to take charge of the subject, and to report at the next congress. Carried unanimously.

Dr. G. S. Norton, of New York, N. Y., read a paper on the

RELATIVE IMPORTANCE OF SMALL DEGREES OF ASTIGMATISM AS A CAUSE OF HEAD-ACHE AND ASTHENOPIA.

He related a number of cases which had come to him, where the correction of astigmatism of only 0.25 D. caused relief to follow with remarkable rapidity. His conclusion was that 0.25 D. of astigmatism may, and not infrequently does, produce disturburbance, which its correction relieves. It most commonly occurs in children and young girls that small degrees occasion disturbance and asthenopia. If the hyperopia is of high degree, it is better to try to correct the trouble with spherical glasses first.

Dr. F. B. Tiffany, of Kansas City, Missouri, next read a paper under the title of

AMETROPIA, introducing a series of statistical tables based on examination of over two thousand school children in Kansas City, Missouri, including three races—white, red, and black. The object of each examination was to ascertain the condition of the eyes, nature of the affection, and endeavor, if possible, to remedy it. The examination took in hypermetropia, astigmatism, and spasm of accommodation, as well as myopia.

The matters of light and ventilation were so well arranged that they might be eliminated as factors in the result. It appeared as if the greater number of ametropes in proportion were in the higher classes, and it might be said that ametropes were more fond of study than emmetropes. Spasm of accommodation developed into myopia. If the eyes were examined each year and corrected carefully, the anomalies would gradually diminish. Hazel eyes are most affected by myopia; blue and gray are stronger than hazel, brown and black. Females have more myopia than males, and Indians are mostly emmetropic.

Professor S. M. Burnett, of Washington, D. C., remarked that he suspected that in the prompt relief spoken of by Dr. Norton the astigmatism was between 0.25 D. and 0.5 D. He found that when one of these cases did so well with 0.25 D., that the total amount was somewhat greater.

Dr. Herbert, of Philadelphia, Pennsylvania, has been astonished to find how little annoyance is felt in high degrees of ametropia and astigmatism, and how much from slight degrees. He had had to not infrequently prescribe as low as 0.2 D.

Dr. R. Tilley, of Chicago, Illinois, remarked on Dr. Tiffany's paper that every new-born child is hypermetropic. Myopia does not generally occur till six or seven years of age.

Dr. H. B. Young, of Burlington, Iowa, remarked that the astigmatic glass determined under atropia was not always accepted. The ciliary muscle did not always readily change its compensatory contraction, and correction by cylinders was sometimes worse than the disease.

Dr. Dickinson, of New York, N. Y., related a case of reflex irritation, with nausea, etc., existing three years, which had been promptly relieved by correction of a very low-power of cylinder.

Dr. Baldwin, of Montgomery, Alabama, wished to state, as to the condition of the negro in regard to refractive troubles, that in over nine hundred cases he had only found an error of refraction in about eight per centum. This seems to bear out the old doctrine that civilization increases eye troubles.

Professor A. Calhoun, of Atlanta, Georgia, stated that it had been his experience that the negro had very little refractive trouble, and if they have not been to school none whatever. He had known of but one near-sighted negro up to ten or fifteen years ago. Now myopia is becoming more frequent. They are subject to other eye troubles, specific affections of the cornea and other parts, but they do not seem to be so serious as in the white, and get well very easily, with almost no treatment at all. Glaucoma is of extremely uncommon occurrence among them. He had only seen three cases among negroes.

Professor Burnett, of Washington, D. C., could corroborate what Dr. Baldwin and Professor Calhoun had said concerning the increase of myopia in the negro of late years, and desired also to call attention to the fact that the negro does dot squint. He thought that glaucoma occurred in the negro, although, perhaps, not to the same extent.

Dr. Galezowski, of Paris, France, said that, in many cases, asthenopia was caused by small degrees of astigmatism, but in many others this had not the effect. He instanced the case of a student, who suffered great inconvenience when using his eyes. Professor Hirschberg, of Berlin, found a slight degree of astigmatism, and corrected it, which relieved the patient for two weeks, and then he became worse again. He came to Paris, and under Dr. Galezowski's notice, who suspected that it was not the ametropia that was causing the trouble. Examination disclosed tenderness over the supra-orbital notch, over the right eye, and all around the left eye. He suspected trouble with the teeth, and found one that had been plugged, but was not tender. He had the plug taken out, and found a small piece of rubber crowded up through the top of the tooth. The tooth was extracted, and the patient recovered. These cases are not infrequent. In other cases it may be a slight alteration in the lachrymal puncture. To test the matter, Dr. Galezowsky injects tepid water, and if it enters the nose badly, it is treated by daily injections and dilatation of the canal, not catheterization.

Professor Reynolds, of Louisville, Kentucky, remarked upon the difficulty of having the patient wear glasses properly, especially in people who were not in the habit of wearing them. In some cases the distance between the eyes has to be so nicely adjusted that the patient must have a pair for distance a little wider apart, and a pair for near work closer together.

Dr. H. Gradle, of Chicago, Illinois, found very few of his cases of asthenopia relieved by weak cylinders.

Dr. Leartus Connor, of Detroit, Michigan, does not recollect that he has had occasion to use as little as 0.25 D.

The president referred to his own experience as corroborating the remarks of the previous speakers in relation to errors of refraction in the negro.

Professor E. Smith, of Detroit, Michigan, found a great amount of refractive error among negroes, and said that it was not uncommon for him to operate upon them for strabismus. It may not be so in the pure negro. In his section of the country they are considerably mixed.

Dr. Erwin, of Ohio, stated that as oculist to a railroad company, he had found that about twenty-five per centum of the men employed in the transportation service are astigmatic, and yet not more than twenty per centum of those referred for special examination have astigmatism. None of the twenty per centum complained of vision, unless referred for spectacles, and only ten per centum of those so referred required correction of astigmatism to relieve suffering.

Dr. Norton, in closing the discussion, said that he had not advanced the subject as anything new. He agreed with Dr. Galezowski that many other causes might produce asthenopia and neuralgic symptoms. As to the exact determination of the amount of astigmatism, he used two sets of radiating lines, one coarser than the other, and when the patient was able to see all the lines in the finer diagram he was quite sure to be accurately corrected.

In adjourning the Section, the President, thanked the members for bearing with him and so cordially supporting his endeavors, and expressed the hope that it had been a season of pleasure and profit to all.

---

### SECTION ON OTOLOGY.

### Fifth Day.

A paper by Dr. B. Loewenberg, Paris, France, was read by title, on

### PHYSIOLOGICAL RESEARCHES ON NASAL VOWELS.

In this paper the author notes first some characteristics of nasal vowels; then certain changes observed in their pronunciation because of adenoid vegetation in the upper air-passages. He gives a table showing comparative pronunciation of the "French nasal vowels," with their equivalents in English and German.

The results of his investigations are given to show how the nasal vowels are formed.

He says: "Amongst the different problems this matter involves arises the question of the musical notes belonging to or characteristic of each nasal vowel. In other terms, we must ascertain which is the sound, or which are the sounds the vocal cavities are tuned to for each of these."

He alludes to similar investigations by Donders, Helmholtz, Koenig, and others regarding "the pure (not nasal) vowels," and says that he adopted methods of investigation similar, in some respects, to some of their modes. The results are given, in tabular form, of the musical notes for each nasal vowel.

Dr. Henry Byrd Young, of Burlington, Iowa, read a paper on

### THE TECHNIPHONE SNAP—A SUBSTITUTE FOR THE WATCH, AS A TEST FOR HEARING.

The insufficiency of the watch as a test for hearing, is well known; and the desirability of something which will more nearly approach that point of excellence reached by Snellen's types as a test for vision, need not be discussed. This ground has been often traversed, and recently again in a very thorough manner by a special committee of the American Otological Society. It is therefore only necessary to say that such a test must be some device which will be simple of construction, cheap, easily managed and reasonably uniform. And combining these conditions very happily and completely is the little device herewith presented and known as the *techniphone snap*—the sounder of the stringless piano called the techniphone.

*It is simple in construction*—consisting merely of a piece of strap steel about ¼m. m. in thickness, 3 c. m. in length, and 1 c. m. in width, dished (dented) slightly at its center in such a way that the edges are not stretched.

*It is cheap.* This is self-evident.

*It is easily managed.* Placing the ends of the strip against the thumb and a finger respectively, preferably of the right hand, the center is siezed edge-wise by the thumb and a finger of the other hand and made to spring against its concavity. When an excursion of about 2 m. m. has been made, the part that is stretched reaches its point of reversal and changes with a distinct snapping sound. Released it springs back, and the sound is repeated. This sound can be produced rapidly, slowly or irregularly as the operator may desire. If the sound is louder than necessary it can be nicely and definitely muffled by siezing the center flat-wise instead of edge-wise—the thumb or finger resting on the point of greatest convexity.

*It is reasonably uniform.* Experience has shown that in the manufacture of these snaps there is no appreciable difference either in pitch or intensity among those having identical measurements. To get a change of pitch there must be a change of dimensions; and other things being equal, the longer strip usually means a lower pitch and the shorter a higher pitch.

Taking it all in all it is a more convenient test than the watch (a stop watch) and, being just as good, should be substituted for it. That it does not fully reach that point of excellence found in Snellen's types as a visual test, is conceded. It is possible to show a relationship between the image of 5 angle and satisfactory vision by a careful computation of the retinal and organs involved. To establish a gauge of such mathematical exactness for the hearing power a better knowledge of the acoustic nerve and its physiology is yet required. If, however, it is borne in mind that the test is not intended to decide how much a patient *should* hear, but merely how much is heard at one or another time; and that with trifling expense and little trouble the patient may be subjected to a number of tests by snaps of different dimensions with their corresponding differences of pitch, the comparison with Snellen's types is not unwarrantable. One great advantage which it possesses over the watch is to be noted in the possible improvement of case reports; and for this, if nothing else, it should be generally adopted. How often, for instance, is it seen recorded that a patient's hearing "as tested by the watch" was $\frac{20}{50}$ in the diseased ear? And what real information is to be gotten from the statement? It is supposed, of course, that the patient heard at twenty inches only, that which had been heard at fifty. But the reader is uninformed as to the character of the sound and is left to wonder whether the hearing must be extraordinarily acute generally, or particularly susceptible to such sounds to perceive it at the greater distance.

With a snap of given measurements there goes a sound of definite pitch and intensity; and it becomes unnecessary to try a number of what may be estimated as normal ears (as recently advised with the watch by the Committee of the American Otological Society) in order to get the so-called average maximum distance at which it can be heard. It only needs be noted that the patient heard the snap *free* at such a distance, or muffled at such another distance; and the remote reader testing his own hearing by the same sound may form a judgment accordingly.

In conclusion, the writer desires to say that his attention was called to this snap quite accidently through a personal meeting at the house of a mutual friend, with the inventor of the techniphone. Its adaptability for the purpose now proposed instantly suggested itself. The snap was procured, used, and found so satisfactory that it was deemed proper to call the attention of others to it. It is therefore before you.

Professor E. De Rossi, Rome, Italy. We have not, at present, any accurate tests for hearing, to determine the condition of the power of hearing. Not even the Acoumeter of Politzer is as serviceable as the watch, which we always carry in our pockets, and the human voice. The watch constitutes a very fair test of the perception of sound, and the human voice is practically the most important sound that the patient desires to perceive. Until such time as we have some more nearly perfect instrument, we must rely on the old instruments which have hitherto served us.

Dr. S. O. Richey thought improvement in any affection of the ear should be judged by structural changes, and that the surgeon should not rely on improvement in function, alone, to decide the more healthful condition of the ear. When the structure is made normal, proper functional activity follows. Tests are chiefly valuable in differentiating the parts of the ear affected, and for indicating the final result, by comparison with the original indications by the same test.

A paper by Dr. L. J. Lautenbach, of Philadelphia, Pennsylvania, on the use of NITRO-GLYCERINE IN TINNITUS AURIUM, was read by title in the absence of the author.

He claims that it has, in his hands, proven to be a valuable remedial agent in the treatment of that troublesome symptom and consequence of ear-disease, tinnitus aurium.

In the paper an effort is made to indicate the special conditions in which this remedy will be found most efficient in relieving this symptom; the most important instances being those in which heart complications exist. He prescribes the remedy in pills, giving one-hundredth of a grain at a dose, to be taken once or twice a day.

A paper was read by Dr. G. W. Allyn, Pittsburgh, Pennsylvania, on the PREVALENCE AND THE TREATMENT OF CHRONIC SUPPURATION OF THE MIDDLE EAR.

The author of the paper, after dwelling upon the frequent occurrence of these cases, and the great neglect of them generally, both by patients and physicians, made an urgent plea for more thorough and more scientific treatment of them, especially by physicians engaged in general practice who frequently are called upon to treat them in the acute stage of the affection, when it is most amendable to treatment. He attributes much of the existing indifference regarding them to ignorance, on the part of many patients, of the gravity of many of the cases, and indifference on the part of the physician who often assumes charge of the case with reluctance, which too frequently comes from want of familiarity with true nature and location of the inflammation.

After reviewing the usual methods of treating this affection, which vary with the peculiarities of the individual cases, he bears further testimony of the value of peroxide of hydrogen, as a means of efficiently cleansing the cavity of the tympanum, and of the impalpable powder of boric acid as an antiseptic and as a means of controlling the suppuration process.

After exhibiting some instruments and apparatus designed for examination and treatment of ear cases, which concluded the work of the session, the president thanked the members of the section for the interest manifested in the work during the sessions, both in the character of the papers presented and the discussion of them, and for their cordial co-operation in the effort made to have the work of the section contribute to the material advancement of otology, and he then declared the last session of the section closed.

---

### SECTION ON LARYNGOLOGY.

### Fifth Day—Morning Session.

In the absence of the authors, several papers were read by title. The only one read and discussed was upon

THE ACTION OF THE EPIGLOTTIS IN SWALLOWING, by Carmalt Jones, M. D., London, England.

The old idea, and perhaps the one which is even now most prevalent, is that epiglottis acts like a lid hinged to the base of the tongue, and by a simple backward movement covering the larnx and directing the food into the œsophagus. The author does not believe that it has any such simple action as that. There is always a slight regurgitation of food and liquids as they are grasped by the middle and inferior constrictors, which would be apt to lift up the epiglottis and pass beneath it. As a result of personal experiments, he finds that the epiglottis on irritation folds the sides together so as to grasp the upper part of the larynx. When this spasm occurs one must wait until it relaxes before trying to pass a brush into the larynx, or it will glide over the epiglottis into the œsophagus. He thinks that the backward movement of the tongue in swallowing is, for the most part, theoretical. One can swallow well with the tongue held between the teeth or firmly grasped by the fingers. There is only a slight effort to draw it back. We swallow more through the action of the pharyngeal muscles. The larynx is raised toward the base of the tongue, and the constrictor muscles do much toward closing it. The epiglottis is drawn down by muscular action and moulds itself about the upper part of the larynx instead of acting like a simple lid.

Dr. O'Dwyer, of New York, N. Y., said that in trying to lift the epiglottis for the introduction of the tube he has found that it is often folded about the larynx so firmly as to require a little force to pass the finger beneath its posterior border and lift it up.

Dr. William Porter, of St. Louis, Missouri, mentioned a case where he had removed the greater part of the epiglottis, and the patient had but little difficulty in swallowing. He thinks that the epiglottis is only accessary, and that the sphincters have much to do in closing the larynx.

Dr. C. S. Allen, of New York, N. Y., related the case of a syphilitic patient where the center of the epiglottis had been destroyed down to its base. On irritation, the long, thin borders, which were left with muscular attachment intact, were closely approximated and formed a fair protection to the larynx.

General discussion followed as to the difference in action under irritation and when simply swallowing.

There being no other papers for the morning session, Professor E. F. Ingals, of Chicago, Illinois, explained a very practicable method of obtaining an abundant supply of compressed air. He had a large kitchen-boiler placed in the basement and connected with the running water, which had a pressure of thirty or forty pounds. The water entering the bottom of the tank compressed the air in the upper part. As the air was used he would allow the water to run into the waste-pipes and air enter the tank from above, and then turn on the water-pressure again.

Dr. C. Slover Allen, of New York, N. Y., presented to the Section

A NEW SNARE AND ECRASEUR.

The advantages of the instrument were shown. It can be used with one hand, even when the greatest power is applied, while the other hand is free for holding the speculum. It is made of steel throughout, so there is no possibibility of its breaking. The ends of the wire pass beneath a small eccentric, and are quickly fastened or released by the simple movement of a lever. The angle of the instrument is such that no part of it or of the hand interferes with a clear view of the part operated on. The rings for middle and index fingers are attached to either end of a double lever, crossing the sliding frame and pivoted to it at the centre. The lever carries a ratchet-catch on either side, which fits into ratchet teeth on the sides of the square rod or frame. The thumb-ring is at the end of this rod. When both finger-rings are pressed upon, the sliding frame is drawn down and held by the ratchets, and the wire loop suddenly contracted upon the tumor. The alternate movement of index and middle fingers causes the advance of the ratchet on one side while the other holds, and the wire is drawn down great leverage power that will cause it to cut through dense tissue.

---

### Afternoon Session.

A paper was read on

THE LONGITUDINAL TENSION OF THE VOCAL CHORDS, ITS PHYSIOLOGY, AND ITS DERANGEMENTS, by Dr. C. M. Desvernine, Havana, Cuba.

The paper was the result of a long series of experiments and dissections, showing the isolated action of each separate muscle, and the effect on the vocal chords where different groups acted as co-ordinate factors. It was exhaustive in treatment, the subject of both in the experiment on the cadaver and upon the action of the muscles in the normal larynx of a living subject. The paper showed the result of hard work, and good, earnest investigation.

TREATMENT OF CHRONIC STENOSIS OF THE LARYNX AND TRACHEA BY INTUBATION, was the title of a paper read by Doctor J. O'Dwyer, of New York, N. Y. He first gave a short sketch of his earlier experiments, and passed around the Section specimens of his tubes showing the gradual improvement from the crude, round and oval short tubes first used to the perfected ones now used. Specimens were shown of the larynx of children of different ages, with the tube inserted. Two specimens were shown of a healthy larynx and diphtheritic one of same age, showing by sections through them at various points that the diminishing of the calibre was caused, not by the false membrane alone, but by a considerable amount of submucous thickening.

The objection had been made that the calibre of the tubes for children was too small, but it was easy to demonstrate that the calibre of the small tubes was larger in proportion to the size of the trachea than was that in the adult tubes. A complete set of the instruments was shown, and the method of introducing and removing the tubes was demonstrated.

The histories of a series of cases were read where subglottic stenosis was successfully treated after tracheotomy had failed. In the first case he was forced to begin with the smallest child's tube, and gradually increase the size until free breathing-space was produced, in eighteen days. The stenosis was the result of syphilitic contraction, and returned in three months; used tubes for a month, and then resorted to occasional introduction for a few hours. Now teaching her to introduce them herself. When worn for a very long time, the metal tube may cause ulceration, which is not the case with rubber. Often finds so much pain and irritation for first few hours after introduction, that he had to give hypodermic injection of morphia, in spite of previous application of cocaine.

He treated a case where syphilitic ulcers had caused adhesion of the chords and complete closure of glottis. Tracheotomy had been performed. He forced a passage through with forceps and finger, and then used tube, but was finally obliged to remove portion of the chords. Cartilaginous support was absorbed and weakened to such an extent that he could not leave out tube. Patient disappeared for ten months with tube still in. When he removed it on her return, found obstruction by granulation tissue. This the tube. Some at first have great difficulty in swallowing while the tube is in the larynx, but soon learn how to do it with more comfort. Semi-solids are better than either solids or liquids.

In tracheal contraction and stenosis he would be inclined to open the trachea and lay in a tube with an enlarged portion or shoulder at each end, and then sew up the wound, leaving it there permanently. The trachea is far less sensative than the larynx.

Dr. L. Browne, of London, England, spoke in the highest terms of the grand success that had attended the years of study and patient work, and said that he was commissioned to purchase a complete outfit for his hospital. He was surprised that tubes could be retained in so sensative an organ.

Dr. O'Dwyer closed the discussion, and exhibited a laryngeal speculum and a snare for removal of membrane and small papillomata from below the vocal chords.

Dr. William Porter, of St. Louis, Missouri, read a paper in which he narrated three cases of benign tumors of the pharnyx. In the second part of the paper he presented a new treatment of cystic goitre, by substituting for the usual iodine injection a piece of catgut steeped in iodine, withdrawing it after inflammation had been excited. The patient wore the compress and did not stop work.

### SECTION ON DERMATOLOGY AND SYPHILOGRAPHY.

FIFTH DAY.

Professor A. R. Robinson, the President, read a paper on

ALOPECIA AREATA, WITH DEMONSTRATION OF DEEP-SEATED MICRO-ORGANISMS.

Believers in the parasitic origin of alopecia have advanced clinical facts to strengthen their theory, while those who believe in its neurotic origin, and they are in the majority, advance other clinical features to substantiate their views. Thus the circular nature of the patches, their appearance shortly after injury of the tissues, nerve-shock, neuralgias, etc., and the occassional spontaneous regrowth of hair. On the other hand, the believers in a fungus or micro-organism refer to the well-known tendency of the disease to spread by continguity of tissue, its occassional occurrence in members of the same family, etc. To prove either case it would be necessary, in the first instance, for the bacteriologists to make cultures, and by inoculation reproduce the disease. In the same way, those who claim a nerve-origin would be forced to show under the microscope changes in the nerve-tissue, or be able to produce a case of alopecia areata by causing an injury of a nerve supplying the scalp. The author carefully and exhaustively reviewed the literature bearing upon the subject of etiology in alopecia, and gave the observations of Eichhorst, Thin, Von Lehlen, and others who have observed micro-organisms in this disease. He called especial attention to the manner of origin and spread of the disease, the absence of inflammatory symptoms, the complete falling out of the hair, etc. If the disease depends entirely upon an organism which is so difficult to find, we should not expect so rapid and sudden a falling of the hair. The complete falling of the hair at once is against this theory of a micro-organism, as some hairs would be likely to escape its ravages and remain scattered over the patch. Again, if so abundant as to cause this, they should have been long since easily detected. The fact of broken-off hairs free from the fungus at the seat of fracture, the manner of the fall of hair, and the condition of these hairs do not permit of a fungus as a cause. Redness in the patch is an exceptional condition, and not to be suspected of being due to a parasite. The loosening and falling out of the hair have been attributed by Dr. Thin to growing organisms. The fibrillation of the hair is the result of atrophy and not of direct action. The hair-root is usually thin and pointed.

Von Schleu discovered organisms especially in the hair-follicles and mostly in the hairs retaining their root-sheaths after epilation. We learn, then, from many observers that micro-organisms are pretty constantly present. Cultures, however, fail. Similar organisms are found upon the normal scalp. Cultures when inoculated produce only slight inflammation. If the disease is parasitic, parasiticides should cure it, while in fact they are not successful in controlling it. The disease is not contagious in the usual sense of the word. Experiments in inoculation are always negative, but too much stress must not be placed on this failure. The liability of the disease to disappear spontaneously interferes with the accurate observation of the effects of remedies. It may be that the failure of parasitic remedies is due to the disappearance of the organisms before the clinical aspects of the affection really appear, or very soon after the hair has fallen. He has found parasiticides of more benefit when employed early, and they are the most trustworthy means at our command at the present time. Dr. Robinson has been led to believe that the disease does not depend upon a micro-organism to be found upon the surface of the skin or in a half-follicle, and has been led to look for the cause in deeper parts. To be sure, alopecia has been produced by injury, and neuraligia frequently precedes or accompanies it—but the observations are too few. A general alopecia from shock should have more importance attached to it. Still there are clinical points against the neurotic theory. The author then described the appearances presented by miscroscopic sections which he had made deep into the tissues, at various stages of the disease. Skin taken from the scalp, on which an alopecia had existed for one week, showed normal epidermis, signs of inflammation in the corium round-cell collection in the sub-papillary layer, cellular infiltration of round-cells blood-vessels dilated, and small arteries contuined fibrinous coagulum. The lymph-channels in the corium were enormously dilated and contained also a fibrinous coagulum. The sebaceous and sweat-glands were not affected.

Some hair shafts showed signs of splitting up and of malnutrition. There was a mild inflammatory condition of the sub-papillary corium, a falling out of the hair, which was replaced by lanugo. In this case the hair grew rapidly again. In a case which had lasted for six months, attributed by the patient to mental disturbances, the same changes were found, but greater ones in the papillary layer. The embryonic cell-collection was marked, and the coagulation distinct. In a case in which the alopecia had existed for several years, causing almost complete baldness, there was found atrophy of all the structures, excepting the vessel-walls. There was some atrophy of the fat-tissues, and only the remains of sebaceous glands were at times found. The hair-follicles showed signs of falling of the hair, there was atrophy of the papillæ, and inflammation, not particularly perifollicular. The inflammatory changes in these cases pointed to this as a direct cause of the loss of hair. In severe cases the scondary disappearance of the sebaceous glands occurs at times and the skin becomes thinner. There is a temporary narrowing of the blood-vessel lumen in all cases. The sudden falling of the hair would be explained by the thickening of the walls and coagula in the vessels supplying the affected area. The inflammatory changes leading to temporary or permanent baldness is a disease of the corium and not of the hair-structures. Alopecia areata is not a hair-disease at all. The author could not consider the disease a trophoneurosis. And the view that it was a vaso-motor disturbance of central origin was not to be entertained, hence he looked for a local cause and found micro-organisms like those described by Von Lehlen. They were present in the lymph-spaces of the corium and sub-papillary layer, with some in the papillæ, and also deep down in the corium. They consisted of cocci in masses, colonies, and lines, and in rows in the lymph-spaces. Diplococci were frequently seen. In the case of one week's duration micrococci were very abundant.

The question arises : Are they accidental or causative ? A local agency is suggested : The spread of a patch ; the development of new ones. The manner of spreading is cleared up by considering micro-organisms causative when we remember how they travel in the lymph-channels as in erysipelas. The slight or non-contagiousness is explained by their deep seat, as is also the frequent failure of parasitic remedies. Treatment must be by penetrating remedies, by internal ones. The soil must be rendered unfavorably by building up a run down subject, or the organisms must be killed directly. Remedies advised are sulphur, pyrogallol, mercury, chrysarobin, which latter is quite reliable. Croton-oil and remedies producing inflammation almost always control it.

In the discussion, Dr. Unna, of Hamburg, Germany, said that just as summer changes to winter and back again, so do the views upon the etiology of alopecia first favor the neurotic then the parasitic theory.

He himself might be said to hold both views. One must, however, have his mind directed to the possibility of a parasitic origin when one sees, as he has, a father and daughters in one family simultaneously affected. On the other hand, an alopecia following an injury to the scalp, as from a needle broken off in the tissues, rather favors the opposite view, and one must recognize a neurotic origin for cases of general alopecia following shock, etc. He could only offer his congratulations to Professor Robinson on the scrupulous original work which he had done and the admirable manner in which he had presented the result of his investigations. The two theories rise side by side, each having points of strength and weakness. He thought it would be a long time before either became the recognized one for all cases of alopecia areata. To prove the parasitic theory, isolation, cultivation, and tranmission were necessary.

Dr. Ravogli, of Cincinnati, Ohio, said he had found small corpuscles resembling micro-organisms in the epidermis, around the hairs, and in the hair-follicles and hair-shaft. He related the case of a lady who complained of an itching in a circumscribed spot, shortly after which a spot of alopecia areata occurred at this point. He related the clinical histories of several other cases.

Dr. Knaggs related a case of general alopecia following a severe dental neuralgia. The perchloride of mercury, pilocarpine in large doses, the extraction of the teeth were without avail. Faradism finally resulted in a cure. After this the patient had marked anæsthesia of the whole body, especially of the scalp. A strong current was necessary to make him feel the application. Afterward he suffered intense agony when the electricity was given, even in weak current.

In other cases he had used twenty per cent. oleate of mercury with benefit.

Dr. J. Zeisler, of Chicago, Illinois, agreed with Dr. Unna, that it would take a long time to bring about a union of the two opinions regarding the cause of alopecia areata. We must admit the presence of micro-organisms when, as now, they after careful methods of staining, etc. Each one must form an opinion for himself, and this opinion is apt to be on the side to which the majority of his own cases appear to belong. He related two cases in which the neurotic origin appeared unmistakable. One was in a case of exopthalmic goitre (Basedow's disease), the other followed a stroke upon the head.

Professor H. J. Reynolds said he had favored the neurotic-origin, but we cannot question Professor Robinson's observations. The cause seems to have been shown by him to reside in the deep skin and the pathology established, but he is not convinced that a neurotic trouble may not give rise to the changes described. It is a question whether the organisms enter from without, or find their way to the locality of the patch from within the body.

He had never seen micro-organisms present. He suggested the use of a galvanic current to aid absorption of parasiticides. This he thought would show whether organisms were the cause of the disease if a cure was hastened.

Dr. Thin said it was evident that we now had a demonstration for the first time of micro-organisms in the deeper tissues of the skin in alopecia. He had come to the conclusion, many years ago, that a blooming fungus about the hair-roots was nothing more than epithelial structures. He had himself described micro-organisms in and around the hair-shafts. After his observations, however for a time the theory of a trophoneurosis predominated.

Clinically, the patients are healthy, and present no cause for attributing the condition to a micro-organism. Since then aniline dyes have come in and Von Schleu has shown a micro-organism. He wished to refute the idea that micro-organisms are present in all hairs.

If the surface of the scalp be disinfected and the epilating forceps scrumpulously clean, none will be found in health. In alopecia they exist in numbers proportionate to the distance from the centre of the patch, few being found at the edges.

He did not think cultivations and reproduction necessary before we could accept the theory. We cannot expect mathematical demonstration in these matters. We accept other organisms as causative. No one doubts the tricophyton, still he is not aware that anyone has succeeded in reproducing ringworm from cultures.

The organism shown resembles the one he saw both in form and size, and he thinks Von Schleu's organism the same. It is not found in the broked hairs, and he thinks the staining and washing washes out many of the organisms. Examination before staining shows minute bodies which have more or less disappeared after washing.

More or less hairs are swollen and ruptured; why should they be unless a force is exerted from their interior?

Professor Robinson's arguments appeal to gentlemen whose minds can accept micro-organisms, for lupus, tuberculosis, lepra, etc. The theory is gradually gaining ground.

Professor Robinson, in closing, said he was sorry more notice had not been taken in the discussion of the histological changes. Inoculation, if practiced, would lead us wrong if a negative result was obtained.

Cases following injury might be explained by our knowledge of the inoculation in osteo-myelitis, the disease not showing itself until the bone has been injured. The existence of organisms in the lymph-spaces explains the disease to his mind, and he is as much convinced as he is that tinea versicolor is a parasitic disease.

---

### SECTION ON CLIMATOLOGY AND DEMOGRAPHY.

### Fifth Day.

The papers read in the section were: "The Demographic Effects of Introduced Diseases, and Especially Leprosy, upon the Hawaiian Races," by Dr. George W. Woods, surgeon, United States Navy; "The Native Treatment of Disease in Syria," by Professor Thomas W. Kay, Syrian Protestant College, Beirut, Syria; "Demographic Consideration of the Evils of Artificial Methods of Preventing Fecundation and of Abortion in Modern Times, by Dr. Thomas M. Dolan, of Halifax, England.

The following is a summary of the paper by Doctor Woods:

Physically and mentally, the Hawaiians were considered superior to all other Polynesian races. They are described as tall, broad-chested, sinewy rather than muscular, with lively, expressive faces, noses slightly flat and often aquiline, mouth and lips large, splendid teeth, and with bodies tattooed. They were intelligent, energetic, kind, simple and hospitable.

Civilization represented, principally by the Anglo-Saxon race, to change or modify the ancient conditions and ways, to impress upon this peculiar people their laws, religion, manners, customs, languages, government, opinions and vices. Climate, soil and general environment remained the same. Hence the altered conditions in their habits and implanted germs of disease must be looked to for an explanation of the astonishing demographic changes which a century has wrought in the Hawaiian race.

The first effect of the contact with civilization was the inoculation of the race with syphilis, which spread so rapidly that it is now often declared that the great majority of the adult population is contaminated. Then came epidemics of scarlatina, rubeola, purtussis, influenza and variola, slaughtering thousands; and the native population, which a century ago had been estimated at four hundred thousand, was reduced to forty thousand in 1884. The extraordinary mortality had been attributed to cachectic conditions, venereal disease, poverty, bad food, licentious and vicious indulgences, including ava-drinking, opium-smoking, and excess in intoxicating liquors. Later, leprosy was introduced by the immigration of the Chinese. This disease was spread rapidly by the good nature, social tastes and hospitality; no fear of the disease, eating from the same dish, and passing the pipe or ava-cup from mouth to mouth; cohabiting with infected persons, and promiscuous and compulsory vaccination.

The first case of leprosy in a native was recogized in 1848. From the establishment of the leper asylum, on the island of Molokai, in 1865 to 1885, three thousand and seventy-six lepers were received, showing in a measure the great prevalence of this scourge. For the eradication of leprosy the following measures are recommended: Segregation of the infected, cremation of the dead bodies and, so far as possible, all discharges, morbid and fæcal, as well as the clothing and bedding, and proper disinfection of the walls, floors and utensils used by the living.

---

### SECTION ON DENTAL AND ORAL SURGERY.

### Fifth Day—Morning Session.

A clinic was given by Dr. A. R. Starr, of New York, N. Y., in capping the exposed pulp of a tooth.

It is only of late years that any progress has been made in so treating and protecting an exposed tooth pulp so as to preserve its vitality. The operation caused the patient very little pain, and all those who have had the opportunity to see it agreed that it was a success.

Dr. William J. Younger, of San Francisco, California, implanted a tooth. Great diversity of opinion was expressed as to the ultimate result of this method of thus supplying the lost teeth.

Dr. R. B. Adair, of Gainesville, Georgia, completed the treatment of a case of pyorrhœa alveolaris.

Other gentlemen gave clinics on filling teeth and constructing artificial dentures.

Professor F. Busch, of Berlin, Germany, read a paper on

THE COMPARATIVE PATHOLOGY OF THE TEETH, WITH SPECIAL REFERENCE TO THE TUSK OF THE ELEPHANT.

A number of specimens were shown by the doctor to illustrate his paper. At the close of his paper the doctor exhibited an instrument for removing the small birth-marks so often found on the face. The instruments have a circular cutting edge ranging in size from one-fourth to half an inch in diameter, and fit into the dental engine. The mode of operation consists in selecting a knife the size of the mark to be removed, and placing it upon it, quickly revolving it. It will take only a moment to accomplish this, and is said to be almost painless. The only dressing he applies is dry cotton, which he leaves in position for from six to eight days, at the end of which time the part is healed.

Dr. E. Andrieu, Paris, France, read a paper on

THE SIXTH YEAR MOLAR.

He held that the six year molar being in development, eruption, and structure an organ of transmission from the temporary to the permanent set, it should be extracted when the permanent teeth are in position. More room is gained by this procedure, and a longer period of usefulness insured to those remaining.

Dr. L. D. Sheperd, of Boston, Massachusetts, expressed his regret that such sentiments should still be prevailing among the profession. He cited cases from practice, when the extracation of these teeth caused not alone loss of valuable masticating surface, but actual irregularity of the ones remaining.

Dr. Paul Dubois, of Paris, France, stated that he wished the Section to note that the ideas expressed in the paper were not carried out by the dentists in France.

Professor Frank Abbott, of New York, could see no difference in structure between the six year molar and the other permanent teeth under the microscope, but a very marked difference was apparent between that and the temporary teeth.

A paper by Dr. Th. David, of Paris, France, on "Aphthous Stomatitis," was read by title.

Professor J. S. Marshall, of Chicago, Illinois, read a paper on

OPERATION FOR THE CURE OF A PERSISTENT NEURALGIA OF BOTH TEMPORO-MAXILLARY ARTICULATIONS, AND REFLECTED PAIN IN THE RIGHT BRACHIAL PLEXUS.

History.—Patient, female, forty-two years of age, was operated on some eight years before for the removal of an osteo-sarcoma of the right inferior maxilla. Extensive suppuration followed, and the wound did not heal for several months. Considerable cicatricial tissue was formed, and the jaw displaced to a considerable extent. Neuralgia dated from the healing of the wound, and the conclusion arrived at was that the irregular position of the jaw was the cause. An operation for the relief of this condition was performed, and the neuralgia ceased. He described the steps of the operation.

A feature of the operation was the appliance used for holding the parts of the jaw in their normal position. This was secured by means of a gold rod screwed into the ramus and attached to the remaining, both on the right side, by a gold crown. This was worn by the patient with no inconvenience for over fifteen months.

Attempts were made to close the gap between the ends of the bone caused by the resection, by means of bone grafting, and which were partially successful, over one inch of new bone having been produced by the grafts, but perfect union was not secured.

Dr. W. H. Atkinson, of New York, N. Y., was requested to open the discussion. He complimented the author on the manner in which he had conducted the case, and expressed appreciation of the suggestions made in reference to bone-grafting and its possibilities. He had no word of adverse criticism, and the Section was honored in having such a paper presented.

The following papers were read by title: "Articulation of Artificial Yeeth," by Dr. H. L. Cruttenden, of Northfield, Minnesota; "Power in Dentistry," by W. St. George Elliot, M. D., of London, England; "Porcelain Filling," by Dr. E. C. Moore, of Detroit, Michigan.

Dr. Alton H. Thompson, of Topeka, Kansas, read a paper entitled

DOES FUNCTION CONTROL THE EVOLUTION OF STRUCTURE?

Some time since, Dr. C. N. Pierce discussed a subject similar to that at the head of this paper. After noticing the mechanical forces involved in and influencing the evolution of the teeth, he says: "Those cumulative forces are utilized through heredity, and while so potent in tooth evolution, exert a similar influence in the development and modification of all other structures and organs. All departments of biology recognize the fact that heredity, adaptation, and growth, being of special importance in the evolution of the organic body, must therefore be regarded as especially formative functions. Adaptation to environment might be called the ancestor of function, as function is of organization. Illustrations of modification of structure in responce to function are very numerous, in which it is shown how an organ may be completely changed or a mere rudiment be fully developed by the demand for the performance of a function unknown before."

A century ago Lamarck laid down the following laws concerning the development of organic life, which we, with all our accumulated facts, can change but little. In his second law he said that, the production of a new organ in an animal body results from the supervention of a new want continuing to make itself felt, and a new movement which this want gives birth to and encourages. Third law: The development of organs and their force of action are constantly in ratio to the employment of those organs. Fourth law: All that has been acquired, laid down, or changed in the organization of the individual, in the course of this life, is commenced by generation and transmitted to new individuals which proceed from those which have undergone those changes." Altered wants lead to altered habits, which result in the formation of new organs, as well as in modification and growth of those previously existing.

In our study of the subject, we will confine our observations to that field in which we, as dental and oral specialists, are most interested the teeth.

Everything is made for a purpose, and the purpose must precede the thing made for effecting the purpose. Nature plans her work as deliberately as man plans his actions. The means for accomplishing an end are not the cause of execution, but the effect. The organ is the effect, and function is the cause of structure. As food-selection is the cause of the function, so the function of the acquisition and preparation of food is thus the cause of the masticating apparatus.

If function is the cause and support of structure, if an organ develops or atrophies as it is used or disused, if the impulse of active employment dictates the evolution of parts and tissues in succeeding generations, if organs have been suppressed through disuse or remain in various forms as mere rudiments—their function have passed away—then, must the teeth of man be tending toward final and inevitable suppression.

If in the future of physical education there shall be found a place for education in the accomplishment of masticating food, then, may the teeth be improved and developed by exercise, as the muscles and lungs are improved and developed.

Owing to the time for adjournment having arrived, no discussion was had on this paper.

The President congratulated the members on the interest they had shown throughout the session, and hoped great good might come from this congress, both to the profession and their patients.

---

GENERAL SESSION.

SATURDAY, SEPTEMBER 10TH—SIXTH DAY—FINAL SESSION.

The Congress was called to order by the President at 9.30 A. M.

The Secretary General reported resolutions from the Section in Climatology, concerning the establishing of a bureau of vital statistics; from the Section in Military and Naval Surgery and Medicine, with regard to the prohibition of the use of explosive balls in warfare; and from the Section in Public and International Hygiene, with reference to the necessity of teaching hygiene in the public schools.

Professor Graily Hewitt, of London, on the part of the foreign members, and after making a few preliminary remarks, in which he conveyed their grateful thanks for the attention which the Congress had received, and their high appreciation of the success which had attended its work, offered the following resolution:

*Resolved*, On the part of the foreign visitors and officers of the Congress, that we desire to convey to the President of the United States our best thanks for his presence at the ceremony of the inauguration of this Congress; that we desire to express to the Executive Committee of the Congress, particularly to Dr. Henry H. Smith, Dr. John B. Hamilton, Dr. A. Y. P. Garnett, Dr. Toner, and Dr. Arnold, our very high appreciation of the efforts they have made for the efficient organization, action, and a working of the Congress, which have resulted in so great a success, and that we would convey our warmest thanks to the citizens of Washington for the kind hospitality, both public and private, we have received during our pleasant visit to their beatiful city.

Dr. Martin, of Berlin, spoke in German, and expressed his thanks for the kindness that had been manifested to him and to the other foreign members of the Congress. He had come here with some doubt, but that had been all removed, and the great success would secure for it high rank among the medical congresses which had been held.

Dr. E. Landolt, of Paris, France, spoke in French, and said that he had been commissioned to express to the President of the United States their sentiments of profound gratitude.

The sanction given by President Cleveland to our Congress has given the greatest charm to our stay in this capital. Returning to our hearths we will preserve the most grateful and respectful remembrance of the President of the Republic, and we say to the whole world that the United States, already so favored, possesses, above everything, a chief who directs them surely in the way of progress and prosperity.

Mr. Edmund Owen, of London, England, seconded the resolution presented by Mr. Hewitt, in a neat speech, which was received with ringing applause. When the history of this grand country is written we trust President Cleveland will have a niche in the temple of fame side by side with those great men, Lincoln and Garfield. When you are in our country we love to hear you say, "We admire your Queen." We can say to you with all truth, we admire, we love your Queen of beauty and of grace, and in seconding this vote of thanks we simply express a prayer that Mr. President Cleveland and Mrs. Cleveland may long continue in strength and health to preside over a happy, a prosperous, and united country.

The Secretary-General, Dr. John B. Hamilton, returned his warmest thanks, especially to the foreign members, who had contributed by their presence, their writings, and their kind words to the success of the Congress.

The President of the Congress, Professor N. S. Davis in the name of the medical profession of the United States, thanked the foreign members for their sympathy and their assistance in the work of the Congress, in which representatives from every State in the Union had been present to greet them, and then declared the Ninth Intertinaonal Medical Congress adjourned.

